# Supplementary material for: Self-reported sleep duration and napping, cardiac risk factors and markers of subclinical vascular disease: cross-sectional study in older men
Source: BMJ Open. 2017 Jul 2;7(6):e016396. doi: 10.1136/bmjopen-2017-016396 (PMC5726087; doi:10.1136/bmjopen-2017-016396)
Supplement: Author's manuscript [file bmjopen-2017-016396.draft_revisions.pdf]

BMJ Open

Self-reported sleep duration and napping, cardiac risk factors and markers of subclinical vascular disease: cross-sectional study in older men

Journal:	*BMJ Open*
Manuscript ID	bmjopen-2017-016396
Article Type:	Research
Date Submitted by the Author:	14-Feb-2017
Complete List of Authors:	Zonoozi, Shahrzad; UCL, Department of Primary Care & Population Health<br>Ramsay, Sheena; Newcastle University, Institute of Health & Society<br>Papacosta, Olia; University College London,<br>Lennon, Lucy; UCL, Department of Primary Care and Population Health<br>Ellins, Elizabeth; Swansea University, Institute of Life Sciences<br>Halcox, Julian; Swansea University, Department of Cardiology<br>Whincup, Peter; St George's University of London,<br>Wannamethee, Goya; UCL, Primary Care and Population Health
**Primary Subject Heading**:	Epidemiology
Secondary Subject Heading:	Cardiovascular medicine
Keywords:	Sleep patterns, Cardiac function, Subclinical atherosclerosis

SCHOLARONE™ Manuscripts

Self-reported sleep duration and napping, cardiac risk factors and markers of subclinical vascular disease: cross-sectional study in older men

Shahrzad Zonoozi[1]; Sheena Ramsay[1]; Olia Papacosta[1]; Lucy Lennon[1]; Elizabeth A Ellins[2]; Julian P J Halcox[2]; Peter H Whincup[3]; S Goya Wannamethee[1]

[1] UCL Department of Primary Care & Population Health, UCL Medical School, Rowland Hill Street, London, NW3 2PF, UK

[2] Institute of Life Sciences, Swansea University, Singleton Park, Swansea, SA2 8PP, UK

[3] Population Health Research Institute, St George's University of London, Cranmer Terrace, London, SW17 0RE, UK

Corresponding author:

Shahrzad Zonoozi, UCL Department of Primary Care & Population Health, UCL Medical School, Rowland Hill Street, London, NW3 2PF, UK. Email: shahrzadz@gmail.com

Word count: 3551; 4 tables

Keywords: sleep patterns, cardiac function, subclinical atherosclerosis

For peer review only

For peer review only - http://bmjopen.bmj.com/site/about/guidelines.xhtml

Abstract

Study Objectives: Daytime sleep has been associated with increased risk of cardiovascular disease and heart failure (HF) but the mechanisms remain unclear. We have investigated the association between daytime and night-time sleep patterns and cardiovascular risk markers in older adults including cardiac markers and subclinical markers of atherosclerosis [arterial stiffness and carotid intima-media thickness (CIMT)].

Methods: Cross sectional study of 1722 surviving men aged 71-92 examined in 2010-2012 across 24 British towns, from a prospective study initiated in 1978-1980. Participants completed a questionnaire and were invited for a physical examination. Men with a history of heart attack or HF (n = 251) were excluded from the analysis.

Results: Self-reported daytime sleep duration was associated with higher fasting glucose and insulin levels (p = 0.02 and 0.01 respectively) even after adjustment for age, BMI, physical activity and social class. Compared to those with no daytime sleep, men with daytime sleep >1 hour, defined as excessive daytime sleepiness (EDS), had a higher risk of raised N-terminal pro brain natriuretic peptide (NT-pro BNP) of ≥400pg/ml, the diagnostic threshold for HF [OR (95% CI) = 1.88 (1.15, 3.1)], higher mean troponin (cTnT), reduced lung function (forced expiratory volume in 1s) and elevated von Willebrand factor (vWF), a marker of endothelial dysfunction. However, EDS was unrelated to CIMT and arterial stiffness. By contrast night-time sleep was only associated with HbA1c (short or long sleep) and arterial stiffness (short sleep).

Conclusions: Daytime sleep duration of >1 hour may be an early indicator of HF.

Word count: 248

For peer review only - http://bmjopen.bmj.com/site/about/guidelines.xhtml

Article Summary

Strength and limitations of this study

- To the best of our knowledge, this is the first study to look at he association between excessive daytime sleepiness (EDS), cardiac markers and vascular parameters.
- Owing to a lack of data, we do not know if the associations seen are true for women, middle aged populations and ethnic minorities.
- As we do not have polysomnography data, we were unable to fully exclude obstructive sleep apnoea as a contributing factor to our findings.
- Our results are based on a cross-sectional analysis and therefore causality cannot be established.

For peer review only

For peer review only - http://bmjopen.bmj.com/site/about/guidelines.xhtml

Introduction

Sleep disturbance is a common complaint, especially amongst older adults. (1) It is increasingly recognised that there is a U shaped association between sleep duration and risk of cardiovascular disease (CVD) and metabolic disturbances – i.e. both short and long sleep durations are associated with an increased risk. (2-4) More recently, studies have shown excessive daytime sleepiness (EDS), one of the more common sleep disturbances reported in the older population, to be associated with increased risks of CVD (5, 6), heart failure (HF) (6, 7) and all-cause mortality. (8-10)

The mechanism of the association between EDS and CVD risk remains unclear. Whilst some studies have looked at the association between EDS and metabolic risk markers (11, 12), few have examined mechanisms by which EDS may influence CVD and in particular incident HF. (6) In particular, the association between EDS and markers of arterial disease and surrogate markers of cardiac function, including NT-pro brain natriuretic peptide (BNP), a marker of ventricular stress, and cardiac troponin T (cTnT), a marker of myocardial injury, have been less studied. Sub-clinical arterial disease markers are increasingly being used as early predictors for atherosclerosis and CVD. A number of non-invasive assessments have been developed, including measures of atherosclerosis such as carotid intima-media thickness (CIMT) and indirect measures of arterial stiffness including carotid distensibility and pulse wave velocity (PWV). Although a number of studies have examined the association between night-time sleep duration and arterial stiffness, (13-17) studies on the association between EDS and vascular risk markers are limited. (5) We have therefore examined the association between daytime sleep as well as night-time sleep with vascular risk factors including metabolic risk factors, inflammatory and endothelial markers, cardiac markers and measures of sub-clinical arterial disease (including arterial stiffness and CIMT).

For peer review only - http://bmjopen.bmj.com/site/about/guidelines.xhtml

Methods

The British Regional Heart Study is a prospective study which recruited a socioeconomically and geographically representative cohort of 7735 men from 24 British towns between 1978 and 1980. The present investigation is based on a reassessment in 2010-2012, when all surviving men (n = 3137), aged between 71–92 years, were sent a postal questionnaire and invited for a 30th year re-examination. 2137 (68%) men completed the postal questionnaire and 1722 (55%) men attended the re-examination. (18) Blood samples were collected after fasting for a minimum of six hours and were stored at −70°C. Ethical approval for data collection was obtained from the relevant research ethics committee. The presence of co-morbidity was based on the men self-reporting a doctor made diagnosis and those with a history of heart attack or HF were excluded from the analysis (n = 251). Of the men with no history of heart attack or HF, 1471 men had physical, blood and ultrasound measurements carried out. This present study is a cross-sectional analysis of the data from the 2010-2012 questionnaires and re-examination.

CVD risk factors: Physical examination included blood pressure (BP), lung function as well as height and weight, from which body mass index (BMI) was calculated. (19) Height standardised forced expiratory volume in 1 second (FEV1) was measured using the Vitalograph Compact II. Details of measurement and classification for smoking status and physical activity in this cohort have been previously described. (20) The use of antihypertensive medication was based on self-reported medication history and participants were also asked to report symptoms of breathlessness. Measurements of metabolic, inflammatory and endothelial markers were taken as described previously. (19, 21, 22) NT-pro BNP and cTnT were measured using the Elecsys 2010 electrochemiluminescence method (Roche Diagnostics, Burgess Hill, UK). NT-pro BNP ≥400pg/ml was defined as high, as per current guidelines issued by the National Institute of Clinical Excellence (NICE) for the diagnosis of HF. (23) Prevalent diabetes included men with doctor-diagnosed diabetes and

For peer review only - http://bmjopen.bmj.com/site/about/guidelines.xhtml

For peer review only

men with fasting blood glucose ≥7 mmol/L. 1402 men had at least one blood measurement (69 missing).

Sleep parameters: Reported duration of night sleep was based on response to: "On average, how many hours of sleep do you have each night?" Duration of day sleep was based on response to "On average, how much sleep (if any) do you have during the daytime?" If a response was entered for the night sleep question but no response was indicated for the day sleep question, the answer was set to 0. 1436 men completed the questions on night-time and daytime sleep duration (35 missing). Night-time sleep was categorised into three groups: <6, 6-8.9 and ≥9 hours. Daytime sleep was categorised into four groups: 0 hours, <1 hour, 1 hour and >1 hour. Those sleeping >1 hour during the daytime were defined as having EDS.

Non-invasive markers of arterial disease: Measurements were measured by two vascular technicians in series. Left and right carotid arteries were images using a Z.One Ultra ultrasound system (Zonare Medical Systems, Mountain View, CA) with a 5-10-mHz linear probe. A cross sectional sweep from the base of the common carotid artery to the jaw bone and longitudinal images of the common carotid artery approximately 1cm proximal to the carotid bifurcation were recorded. Peak systolic and end-diastolic common carotid artery diameter and CIMT, the distance between the leading edge of the intima and the media-adventitia interface, were measured using Carotid Analyser software (Medical Imaging Applications, Iowa City, IA). From the longitudinal images, a region of interest (5-10 mm) was selected in a plaque free area, at least 1cm from the bifurcation.

CIMT was measured from three end-diastolic images on each side and a mean of these measures was calculated. Maximum and minimum carotid artery diameter was assessed from three consecutive waveforms and mean distension was calculated as: maximum diameter – minimum diameter. The distensibility coefficient was then calculated as described by Dijk *et al.*: distensibility coefficient = [(2 x mean distension/baseline diameter)/mean pulse pressure (kPa)]*1000. (24) Carotid to femoral PWV was assessed using a Vicorder

For peer review only

(Skidmore Medical, Bristol UK), with participants in a semi-supine position with their torso at approximately 30˚. A 2x9cm cuff was positioned around the neck with the bladder over the right carotid pulse, and a Hokanson SC10 cuff around the middle of the right thigh. Path length was measured from the sternal notch to the centre of the thigh cuff. The cuffs were simultaneously inflated and traces with a minimum of 3 good quality waveforms recorded. Two PWV measurements, within ≤0.5m/s of each other, were accepted and averaged. 1464 men had at least one non-invasive vascular measurement carried out (7 missing).

Statistical analysis: Distributions of HbA1c, glucose, insulin, CRP, IL-6, NT-pro BNP and cTnT were highly skewed and log transformation was used. Comparisons of baseline characteristics between the sleep groups were carried out using the chi squared test for categorical variables and analysis of variance for continuous variables. Logistic regression models were used to assess the differences in outcomes between categorical groups. In multivariate analyses, age, BMI, physical activity and social class were fitted as continuous variables whilst prevalent diabetes and use of antihypertensive medications was defined as categorical variables. The base groups for the statistical analysis were the group sleeping 6-8.9 hours in the night-time sleep group. A threshold effect was seen in those sleeping >1 hour in the daytime and therefore we compared those sleeping >1 hour with those sleeping ≤1. We also carried out statistical analysis of the trend in the daytime sleep group. All analyses were performed using SAS version 9.3 (SAS, Cary, North Carolina).

Results

The average duration of night-time sleep in the men who responded was 6.8 hours (± 1.4 hours). 15% of the men reported night-time sleep duration of less than 6 hours, while 7% reported sleeping ≥9 hours during the night. 47% of men reported no daytime sleep whilst 9% reported EDS. Night-time sleep duration was significantly associated with EDS (p = 0.004), with a smaller proportion of those sleeping ≥9 hours reporting EDS. Daytime sleep duration was significantly associated with night-time sleep of less than 6 hours (p = 0.02).

For peer review only - http://bmjopen.bmj.com/site/about/guidelines.xhtml

For peer review only

Baseline characteristics

Table 1 describes the baseline characteristics of the study population according to night-time and daytime sleep patterns. A U-shaped relationship was seen between night-time sleep and CVD risk factors. Both short and long night-time sleep were associated with increased age, increased prevalence of physical inactivity, manual workers and breathlessness compared to those with 6-8.9 hours sleep. Daytime sleep is strongly associated with older age and with many adverse characteristics including physical inactivity, obesity, prevalent diabetes, breathlessness, use of anti-hypertensive medication, depression and chronic kidney disease (CKD). The prevalence of these adverse factors tended to increase with increasing duration of daytime sleep.

Night-time sleep and and cardiovascular risk markers

There was a U-shaped association between night-time sleep and metabolic risk factors including HbA1c and glucose (Table 2). In age-adjusted analysis, night-time sleep was significantly associated with IL-6, a marker of inflammation, but this association was attenuated following adjustment for BMI, social class and physical activity (p = 0.05). No significant association was seen between night-time sleep and von Willebrand factor (vWF), a marker of endothelial function, FEV1 (lung function) or cardiac markers (NT-proBNP and cTnT).

Daytime sleep and cardiovascular risk markers

In age-adjusted analysis, metabolic risk factors including HDL-C, triglycerides, HbA1c and insulin tended to increase with increasing hours of daytime sleep (Table 3). No association was seen with BP. The increasing trend was attenuated after adjustment for age, BMI, social class and physical activity. However compared to those who slept ≤1 hour, those with EDS

For peer review only - http://bmjopen.bmj.com/site/about/guidelines.xhtml

For peer review only

(>1 hour) showed significantly higher mean levels of HbA1c, glucose and insulin (Table 3). Mean levels of glucose and insulin remained significantly higher in those with EDS (p = 0.02 and 0.01 respectively) even after further adjustment for prevalent diabetes.

CRP increased with increasing daytime sleep duration, even after full adjustment (p trend = 0.04). Lung function decreased with increasing daytime sleep duration with levels markedly reduced in those with EDS compared to those reporting ≤1 hour daytime sleep (p < 0.0001). Only EDS was associated with significantly higher mean vWF levels (p = 0.03). When we examined the odds of having high vWF (top quintile) and low FEV1 (lowest quintile), those with EDS were 1.8 times more likely to have a high vWF [OR (95% CI) = 1.82 (1.17, 2.83)] and 2.4 times more likely to have a low FEV1 [OR (95% CI) = 2.4 (1.53, 3.74)] compared to those who reported no daytime sleep.

Mean levels of NT-pro BNP and cTnT (cardiac markers) were significantly higher only in those with EDS following full adjustment (p = 0.02 and 0.03 respectively). This association remained significant even after adjustment for CKD. Men with EDS were more likely to have high (top quintile) mean NT-pro BNP [OR (95% CI) = 1.69 (1.06, 2.7)] and cTnT [OR (95% CI) = 1.75 (1.09, 2.8)] compared to those who reported no daytime sleep. However, this was attenuated following adjustment for prevalent diabetes, use of antihypertensive medication and depression [OR (95% CI) = 1.59 (0.99, 2.55)] and [OR (95% CI) = 1.62 (1.00, 2.62)] respectively, although it remained significant for cTnT. We further looked at men with a NT-pro BNP of ≥400pg/ml, the diagnostic threshold for HF. Men who reported EDS had a higher odds of having an NT-pro BNP of ≥400pg/ml [OR (95% CI) = 1.88 (1.15, 3.1)]. This association remained following adjustment for prevalent diabetes, use of antihypertensive medication and depression [OR (95% CI) = 1.73 (1.04, 2.86)].

Sleep patterns and non-invasive markers of arterial disease

For peer review only - http://bmjopen.bmj.com/site/about/guidelines.xhtml

Table 4 shows the associations between sleeping patterns and non-invasive vascular measurements. There was a significant association between those sleeping less than 6 hours and mean PWV and distensibility when compared to those sleeping 6-8.9 hours in the fully adjusted model (p = 0.04 and 0.03 respectively). Men who slept less than 6 hours during the night-time were 1.6 times more likely to have a low (lowest quintile) mean distensibility compared to those who slept 6-8.9 hours in the fully adjusted model [OR (95% CI) = 1.56 (1.09, 2.23)]. There was no significant association between daytime sleep and markers of arterial stiffness. There was no association between sleeping patterns and CIMT in the study population.

For peer review only

For peer review only - http://bmjopen.bmj.com/site/about/guidelines.xhtml

Discussion

We have confirmed previous reports of an association between night-time sleep duration (4) and daytime sleep duration (11, 12) with metabolic risk factors. Our findings extend previous studies by investigating the association between EDS and a wide range of cardiac markers and vascular measures not previously examined. Our study has shown that in a representative sample of British men aged 71–92 years with no prior history of heart attack or HF, those who sleep more than one hour during the day have a number of adverse characteristics including higher mean levels of cardiac markers, metabolic risk factors, inflammatory and endothelial markers and reduced lung function. These findings may point to pathways for the association observed between EDS and CVD and all-cause mortality. (8)

EDS and metabolic and cardiac risk markers

EDS, defined in our study as daytime sleep of >1 hour, is associated with many adverse characteristics such as obesity, physical inactivity, diabetes, breathlessness, use of antihypertensive therapy and with an adverse pattern of metabolic risk factors including HbA1c, glucose and insulin. Our results confirm previous cross-sectional analyses which have shown EDS to be associated with the metabolic syndrome and longer habitual napping duration to be associated with an increased risk of impaired fasting glucose and diabetes mellitus. (11, 12) There was a significant linear relationship between increasing daytime sleep and mean CRP levels, with those reporting EDS having higher levels of mean CRP. This confirms results from a previous study which showed self-reported daytime napping is associated with higher levels of CRP in older British adults. (25) The association seen between daytime sleep and these markers of inflammation may provide an explanation for the excess all-cause mortality observed in those with daytime napping or EDS. (8, 9) Whilst both of these studies have adjusted for diabetes, neither has adjusted for inflammation.

We found a significant association between EDS and high levels of NT-pro BNP and cTnT, both of which are biomarkers of HF. (26, 27) NT-pro BNP is produced in response to cardiac

For peer review only - http://bmjopen.bmj.com/site/about/guidelines.xhtml

wall stress and is a strong predictor of HF. (22) Troponin T is a plasma marker of myocyte necrosis, which has been positively associated with incident CVD, HF and all-cause mortality. (28) Most notably men who reported EDS had a higher odds of having NT-pro BNP of ≥400pg/ml (the diagnostic threshold for HF). As far as we are aware, this is the first study to look at the association between EDS and these cardiac markers.

A strong association was also seen between EDS and elevated vWF (marker of endothelial dysfunction) and low FEV1, markers associated with HF. These findings suggest that EDS may be an early indicator of HF. This supports a recent report from our study when the men were aged 63-82 years which showed EDS to be associated with the risk of developing overt HF during 9 year follow-up. (7) In that analyses cardiac markers and lung function were not available.

The association between EDS and HF may be explained by underlying respiratory illness such as obstructive sleep apnoea (OSA). Indeed within our cohort, EDS was significantly associated with obesity, self-reported breathlessness and reduced lung function. EDS may be a result of disturbed night-time sleep and difficulty falling asleep, both of which have been associated with HF. (29, 30) However, the associations we observed with cardiac markers and lung function were not seen in men with a short or long duration of night-time sleep and further analyses showed no association between these markers and those reporting difficulty falling asleep.

Non-invasive markers of arterial disease

In contrast to night-time sleep we did not observe any significant association between daytime sleep and markers of arterial stiffness. Short night-time sleep but not long night-time sleep was associated with arterial stiffness (PWV and carotid distensibility), when compared to those sleeping 6-8.9 hours. There was no association between sleeping patterns and CIMT in the study population. This is in contrast to previous studies which have shown an

For peer review only - http://bmjopen.bmj.com/site/about/guidelines.xhtml

For peer review only

association between short night-time sleep (17) and EDS (5) with CIMT. The differences observed may be due to use of brachial-ankle PWV (13, 15), rather than carotid-femoral PWV, different classification of long night-time sleep (13, 14) and a younger study population. (15)

Strengths and limitations

The strengths of our study include the fact that it is a study of older men, a group who are at high risk of vascular disease and the wide range of vascular parameters which we have looked into. Limitations of our study include the lack of representation of women, middle aged populations and ethnic minority groups which limits generalisability. Our data is based on self-reported sleeping patterns at a single time point and it is possible that night-time and daytime sleep duration have been misclassified. Our analysis of night-time sleep relies on average duration of night-time sleep and we do not have measures of interruption or total time spent in bed. As we do not have polysomnography data, we were unable to fully exclude OSA as a contributing factor to our findings. We appreciate that our results are based on a cross-sectional analysis and therefore causality cannot be established. It is also possible that there is an element of survival bias in our data; it may be that men who attended the re-examination were healthier and this may account for the null associations seen.

Conclusions

EDS, defined as daytime sleep of more than one hour, is associated with an adverse cardiovascular profile and may be an early indicator of HF. Clinically, this is important in the assessment of older patients who seek medical advice for EDS, in whom indicators of HF should be sought and further investigations carried out if necessary.

For peer review only - http://bmjopen.bmj.com/site/about/guidelines.xhtml

Disclosures

The authors report no conflicts of interest.

Funding

The British Regional Heart Study is a British Heart Foundation (BHF) research group. This work was supported by a British Heart Foundation programme grant (RG/13/16/30528) and project grant (PG/09/024, PG/13/41/30304).

Contributions

SGW initiated the concept and design of the paper. SZ analysed the data and drafted the manuscript. SGW, JPJH, EAE, PHW contributed to the interpretation of data. OP contributed to the analysis of the paper. EAE, JPJH, LL, PHW and SGW contributed to the acquisition of the data. All authors revised it critically for important intellectual content and approved the final version of the manuscript.

Data sharing statement

Further details of the Study can be found on http://www.ucl.ac.uk/pcph/research-groups-themes/brhs-pub. For general data sharing enquiries, please contact Lucy Lennon l.lennon@ucl.ac.uk

For peer review only

Table 1 - Baseline characteristics of the study population

		Age mean ±std	Obese (%)	Cigarette Smoker (%)	Inactive (%)	Manual social class (%)	Diabetic (%)	Breathlessness (%)	Using Antihypertensives (%)	Depression (%)	CKD (%)
**Night-time Sleep Duration (hours)**	**< 6 (n = 213[a])**	78.76±4.6	21.53	0.94	48.36	57.35	18.31	40.72	53.99	11.43	29
	**6-8.9 (n = 1126[a])**	78.22±4.6	18.78	3.83	33.66	42.4	14.87	19.32	48.85	8.39	29.36
	**≥ 9 (n = 97[a])**	79.24±4.9	22.11	4.12	38.14	48.45	19.79	25	52.58	11.34	38.71
	**p**	0.05	0.52	0.10	0.0002 *	0.002 *	0.24	<.0001 *	0.33	0.27	0.16
**Daytime Sleep Duration (hours)**	**0 (n = 680[a])**	77.83±4.4	16.1	2.65	30.74	46.1	12.24	17.59	45.88	7.41	26.42
	**<1 (n = 290[a])**	78.04±4.4	15.63	2.76	33.45	40	14.88	21.61	54.14	10.42	30.94
	**1 (n = 336[a])**	78.79±4.7	24.17	5.07	40.77	44.61	20	27.01	49.7	8.98	32.81
	**>1 (n = 130[a])**	80.05±5	33.33	4.62	58.46	51.54	24.62	41.46	61.54	14.62	39.17
	**p**	<.0001 *	<.0001 *	0.18	<.0001 *	0.15	0.0003 *	<.0001 *	0.004 *	0.05	0.02 *

a, maximum n in group, varies slightly with missing co-variate data

CKD, chronic kidney disease

For peer review only

For peer review only - http://bmjopen.bmj.com/site/about/guidelines.xhtml

Table 2 - Night-time sleep duration vs metabolic risk factors, inflammatory and endothelial markers, lung function and cardiac markers.

		Night Sleep (hours)			
		< 6 (n = 213[d])	6-8.9 (n = 1126[d])	≥ 9 (n = 97[d])	p
**Metabolic risk factors [mean (95% CI)]**					
**SBP (mmHg)**	**A**	146.04 (143.52, 148.56)	147.31 (146.21, 148.4)	150.73 (146.99, 154.47)	0.12
**DBP (mmHg)**	**A**	76.59 (75.07, 78.11)	77.46 (76.8, 78.12)	77.25 (74.99, 79.5)	0.59
**HDL-C (mmol/L)**	**A**	1.47 (1.41, 1.53)	1.48 (1.45, 1.5)	1.37 (1.28, 1.46)	0.07
**Triglycerides (mmol/L)**	**A**	1.3 (1.21, 1.39)	1.28 (1.25, 1.32)	1.39 (1.26, 1.52)	0.31
**HbA1c[c] (%)**	**A**	5.93 (5.85, 6.02)	5.75 (5.72, 5.79)	5.88 (5.76, 6)	0.0001 *
	**B**	5.9 (5.82, 5.98)	5.76 (5.73, 5.79)	5.86 (5.74, 5.97)	0.003 *
**Glucose[c] (mmol/L)**	**A**	5.78 (5.62, 5.94)	5.55 (5.48, 5.61)	5.72 (5.49, 5.95)	0.02 *
	**B**	5.74 (5.58, 5.9)	5.56 (5.49, 5.62)	5.68 (5.45, 5.91)	0.08
**Insulin[c] (mU/L)**	**A**	8.26 (7.54, 9.04)	7.92 (7.62, 8.24)	7.82 (6.84, 8.94)	0.69
**Inflammatory and endothelial markers [mean (95% CI)]**					
**CRP[c] (mg/L)**	**A**	1.51 (1.28, 1.78)	1.34 (1.25, 1.44)	1.46 (1.15, 1.87)	0.37
**IL-6[c] (pg/mL)**	**A**	3.03 (2.74, 3.35)	2.98 (2.86, 3.11)	3.72 (3.21, 4.3)	0.02 *
	**B**	2.94 (2.67, 3.25)	3 (2.87, 3.13)	3.61 (3.12, 4.18)	0.05
**vWF (IU/dL)**	**A**	140.97 (130.97, 150.97)	133.27 (128.91, 137.63)	119.04 (104.2, 133.89)	0.06
**Lung function [mean (95% CI)]**					
**FEV1 (L)**	**A**	2.43 (2.35, 2.5)	2.46 (2.42, 2.49)	2.44 (2.32, 2.55)	0.76
**Cardiac markers [mean (95% CI)]**					
**NT-pro BNP[c] (pg/mL)**	**A**	121.67 (101.33, 146.1)	126.45 (116.87, 136.81)	134.61 (102.8, 176.25)	0.83
**cTnT[c] (pg/ml)**	**A**	11.25 (10.29, 12.3)	10.82 (10.41, 11.24)	10.14 (8.89, 11.57)	0.44

A, adjusted for age

B, adjusted for age, BMI, social class and physical activity

c, geometeric mean

d, maximum n in group, varies slightly with missing co-variate data

SBP, systolic blood pressure; DBP, diastolic blood pressure; HDL-C, high density lipoprotein cholesterol; HbA1c, glycated haemoglobin; CRP, C-reactive protein; IL-6, interleukin 6; vWF, von Willebrand factor; FEV1, forced expiratory volume in 1s; NT-pro BNP, N-terminal pro brain natriuretic peptide; cTnT, cardiac troponin T

For peer review only

For peer review only - http://bmjopen.bmj.com/site/about/guidelines.xhtml

Table 3 – Daytime sleep duration vs metabolic risk factors, inflammatory and endothelial markers, lung function and cardiac markers.

		Daytime Sleep Duration (Hours)					
		0 (n = 680[d])	<1 (n = 290[d])	1 (n = 336[d])	>1 (n = 130[d])	p value trend	p value >1h vs others
**Metabolic risk factors [mean (95% CI)]**							
**SBP (mmHg)**	**A**	147.46 (146.05, 148.88)	148.76 (146.59, 150.92)	147.14 (145.13, 149.15)	144.13 (140.88, 147.39)	0.90	0.04 *
	**B**	147.49 (146.07, 148.9)	148.59 (146.42, 150.75)	146.97 (144.95, 148.99)	144.78 (141.44, 148.11)	0.96	0.12
**DBP (mmHg)**	**A**	77.41 (76.56, 78.26)	78.08 (76.77, 79.38)	77.16 (75.95, 78.37)	75.54 (73.57, 77.5)	0.98	0.06
	**B**	77.56 (76.7, 78.41)	78.04 (76.74, 79.35)	76.94 (75.73, 78.16)	75.64 (73.63, 77.65)	0.74	0.09
**HDL-C (mmol/L)**	**A**	1.5 (1.47, 1.54)	1.47 (1.42, 1.52)	1.44 (1.39, 1.49)	1.33 (1.25, 1.41)	0.01 *	0.0002 *
	**B**	1.49 (1.46, 1.52)	1.46 (1.41, 1.5)	1.46 (1.42, 1.51)	1.41 (1.33, 1.48)	0.14	0.10
**Triglycerides (mmol/L)**	**A**	1.24 (1.19, 1.29)	1.3 (1.23, 1.38)	1.35 (1.28, 1.42)	1.42 (1.31, 1.54)	0.01 *	0.02 *
	**B**	1.26 (1.21, 1.3)	1.33 (1.26, 1.4)	1.33 (1.26, 1.4)	1.34 (1.22, 1.45)	0.03 *	0.47
**HbA1c[c] (%)**	**A**	5.73 (5.69, 5.78)	5.74 (5.68, 5.81)	5.87 (5.8, 5.93)	5.99 (5.88, 6.1)	0.01 *	0.0003 *
	**B**	5.75 (5.7, 5.79)	5.77 (5.7, 5.83)	5.84 (5.78, 5.91)	5.9 (5.79, 6.01)	0.04 *	0.04 *
**Glucose[c] (mmol/L)**	**A**	5.52 (5.43, 5.6)	5.48 (5.36, 5.61)	5.7 (5.58, 5.83)	5.98 (5.77, 6.2)	0.09	0.0002 *
	**B**	5.54 (5.45, 5.62)	5.51 (5.38, 5.63)	5.68 (5.56, 5.8)	5.86 (5.65, 6.07)	0.25	0.01 *
**Insulin[c] (mU/L)**	**A**	7.5 (7.13, 7.88)	7.43 (6.89, 8.02)	8.69 (8.09, 9.33)	10.36 (9.21, 11.65)	0.01 *	<.0001 *
	**B**	7.7 (7.36, 8.07)	7.68 (7.16, 8.24)	8.31 (7.77, 8.88)	9.29 (8.32, 10.37)	0.15	0.005 *
**Inflammatory and endothelial markers [mean (95% CI)]**							
**CRP[c] (mg/L)**	**A**	1.24 (1.13, 1.36)	1.36 (1.19, 1.57)	1.56 (1.37, 1.78)	1.67 (1.35, 2.08)	0.01 *	0.07
	**B**	1.27 (1.16, 1.39)	1.4 (1.22, 1.61)	1.5 (1.32, 1.71)	1.51 (1.21, 1.89)	0.04 *	0.37
**IL-6[c] (pg/mL)**	**A**	2.88 (2.73, 3.05)	3.01 (2.76, 3.28)	3.19 (2.95, 3.45)	3.58 (3.14, 4.09)	0.03 *	0.01 *
	**B**	2.92 (2.76, 3.08)	3.06 (2.81, 3.33)	3.13 (2.89, 3.39)	3.34 (2.92, 3.81)	0.10	0.15
**vWF (IU/dL)**	**A**	132.65 (127.06, 138.24)	127.34 (118.81, 135.87)	134.92 (126.85, 142.98)	148.03 (135.05, 161.01)	1.00	0.02 *
	**B**	132.76 (127.1, 138.41)	127.64 (119.03, 136.24)	134.88 (126.7, 143.07)	147.74 (134.39, 161.1)	0.96	0.03 *
**Lung function [mean (95% CI)]**							
**FEV1 (L)**	**A**	2.52 (2.48, 2.57)	2.46 (2.39, 2.52)	2.37 (2.31, 2.44)	2.21 (2.11, 2.31)	<.0001 *	<.0001 *
	**B**	2.52 (2.48, 2.56)	2.44 (2.38, 2.51)	2.38 (2.32, 2.44)	2.26 (2.15, 2.36)	<.0001 *	<.0001 *
**Cardiac markers [mean (95% CI)]**							
**NT-pro BNP[c] (pg/mL)**	**A**	122.31 (110.53, 135.34)	110.95 (95.15, 129.37)	135.15 (117.08, 156.02)	171.15 (134.5, 217.78)	0.70	0.01 *
	**B**	123.28 (111.4, 136.43)	111.2 (95.39, 129.62)	135.76 (117.52, 156.82)	167.33 (130.92, 213.88)	0.82	0.02 *
**cTnT[c] (pg/ml)**	**A**	10.33 (9.83, 10.85)	10.45 (9.7, 11.27)	11.59 (10.8, 12.43)	12.79 (11.37, 14.38)	0.03 *	0.004 *
	**B**	10.41 (9.91, 10.93)	10.52 (9.77, 11.33)	11.31 (10.55, 12.13)	12.3 (10.93, 13.86)	0.12	0.03 *

For peer review only

For peer review only - http://bmjopen.bmj.com/site/about/guidelines.xhtml

A, adjusted for age

B, adjusted for age, BMI, social class and physical activity

c, geometeric mean

d, maximum n in group, varies slightly with missing co-variate data

SBP, systolic blood pressure; DBP, diastolic blood pressure; HDL-C, high density lipoprotein cholesterol; HbA1c, glycated haemoglobin; CRP, C-reactive protein; IL-6, interleukin 6; vWF, von Willebrand factor; FEV1, forced expiratory volume in 1s; NT-pro BNP, N-terminal pro brain natriuretic peptide; cTnT, cardiac troponin T

For peer review only

For peer review only - http://bmjopen.bmj.com/site/about/guidelines.xhtml

Table 4 – Sleeping patterns vs non-invasive vascular measurements.

		CIMT (mm)		PWV (m/s)		Distensibility ($10^{-3}kPa^{-1}$)	
		Age-adjusted	Additional Adj[a]	Age-adjusted	Additional Adj[a]	Age-adjusted	Additional Adj[a]
		mean (95% CI)	mean (95% CI)	mean (95% CI)	mean (95% CI)	mean (95% CI)	mean (95% CI)
**Night-time Sleep Duration (hours)**	**< 6 (n = 209[b])**	0.81 (0.78, 0.83)	0.8 (0.78, 0.82)	10.48 (10.24, 10.71)	10.45 (10.22, 10.69)	11.66 (11.11, 12.21)	11.62 (11.06, 12.18)
	**6-8.9 (n = 1125[b])**	0.81 (0.8, 0.82)	0.81 (0.8, 0.82)	10.18 (10.08, 10.27)	10.18 (10.08, 10.28)	12.29 (12.06, 12.53)	12.31 (12.08, 12.55)
	**≥ 9 (n = 95[b])**	0.82 (0.79, 0.85)	0.82 (0.79, 0.85)	10.25 (9.91, 10.6)	10.25 (9.9, 10.59)	11.71 (10.9, 12.51)	11.81 (11, 12.63)
	**p**	0.67	0.64	0.07	0.11	0.06	0.06
**Daytime Sleep Duration (hours)**	**0 (n = 677[b])**	0.81 (0.79, 0.82)	0.81 (0.8, 0.82)	10.25 (10.12, 10.38)	10.25 (10.12, 10.38)	12.23 (11.93, 12.53)	12.2 (11.89, 12.5)
	**<1 (n = 290[b])**	0.81 (0.79, 0.82)	0.81 (0.79, 0.82)	10.15 (9.96, 10.35)	10.16 (9.96, 10.36)	12.49 (12.03, 12.96)	12.5 (12.04, 12.97)
	**1 (n = 334[b])**	0.8 (0.79, 0.82)	0.8 (0.78, 0.82)	10.2 (10.01, 10.38)	10.2 (10.01, 10.38)	11.81 (11.38, 12.25)	11.88 (11.44, 12.32)
	**>1 (n = 128[b])**	0.84 (0.81, 0.87)	0.83 (0.8, 0.86)	10.29 (9.99, 10.6)	10.29 (9.98, 10.6)	11.96 (11.25, 12.67)	12.12 (11.39, 12.85)
	**p**	0.17	0.37	0.80	0.85	0.18	0.30

a, adjusted for age, BMI, social class and physical activity

b, maximum n in group

CIMT, carotid intima-media thickness; PWV, pulse wave velocity

For peer review only - http://bmjopen.bmj.com/site/about/guidelines.xhtml

Bibliography

1. Flamer HE. Sleep disorders in the elderly. Australian and New Zealand Journal of Medicine. 1996;26(1):96-104.
2. Grandner MA, Sands-Lincoln MR, Pak VM, Garland SN. Sleep duration, cardiovascular disease, and proinflammatory biomarkers. Nature and science of sleep. 2013;5:93-107.
3. Jackson CL, Redline S, Emmons KM. Sleep as a potential fundamental contributor to disparities in cardiovascular health. Annual review of public health. 2015;36:417-40.
4. Knutson KL. Sleep duration and cardiometabolic risk: a review of the epidemiologic evidence. Best practice & research Clinical endocrinology & metabolism. 2010;24(5):731-43.
5. Blachier M, Dauvilliers Y, Jaussent I, Helmer C, Ritchie K, Jouven X, et al. Excessive daytime sleepiness and vascular events: the Three City Study. Annals of neurology. 2012;71(5):661-7.
6. Newman AB, Spiekerman CF, Enright P, Lefkowitz D, Manolio T, Reynolds CF, et al. Daytime sleepiness predicts mortality and cardiovascular disease in older adults. The Cardiovascular Health Study Research Group. Journal of the American Geriatrics Society. 2000;48(2):115-23.
7. Wannamethee SG, Papacosta O, Lennon L, Whincup PH. Self-Reported Sleep Duration, Napping, and Incident Heart Failure: Prospective Associations in the British Regional Heart Study. Journal of the American Geriatrics Society. 2016.
8. Leng Y, Wainwright NW, Cappuccio FP, Surtees PG, Hayat S, Luben R, et al. Daytime napping and the risk of all-cause and cause-specific mortality: a 13-year follow-up of a British population. American journal of epidemiology. 2014;179(9):1115-24.
9. Tanabe N, Iso H, Seki N, Suzuki H, Yatsuya H, Toyoshima H, et al. Daytime napping and mortality, with a special reference to cardiovascular disease: the JACC study. International journal of epidemiology. 2010;39(1):233-43.
10. Zhong G, Wang Y, Tao T, Ying J, Zhao Y. Daytime napping and mortality from all causes, cardiovascular disease, and cancer: a meta-analysis of prospective cohort studies. Sleep medicine. 2015;16(7):811-9.
11. Hayley AC, Williams LJ, Kennedy GA, Berk M, Brennan SL, Pasco JA. Excessive daytime sleepiness and metabolic syndrome: a cross-sectional study. Metabolism. 2015;64(2):244-52.
12. Fang W, Li Z, Wu L, Cao Z, Liang Y, Yang H, et al. Longer habitual afternoon napping is associated with a higher risk for impaired fasting plasma glucose and diabetes mellitus in older adults: results from the Dongfeng–Tongji cohort of retired workers. Sleep medicine. 2013;14(10):950-4.
13. Tsai TC, Wu JS, Yang YC, Huang YH, Lu FH, Chang CJ. Long sleep duration associated with a higher risk of increased arterial stiffness in males. Sleep. 2014;37(8):1315-20.
14. Abe T, Aoki T, Yata S, Okada M. Sleep duration is significantly associated with carotid artery atherosclerosis incidence in a Japanese population. Atherosclerosis. 2011;217(2):509-13.
15. Yoshioka E, Saijo Y, Kita T, Okada E, Satoh H, Kawaharada M, et al. Relation between self-reported sleep duration and arterial stiffness: a cross-sectional study of middle-aged Japanese civil servants. Sleep. 2011;34(12):1681-6.
16. Wolff B, Volzke H, Schwahn C, Robinson D, Kessler C, John U. Relation of self-reported sleep duration with carotid intima-media thickness in a general population sample. Atherosclerosis. 2008;196(2):727-32.
17. Nakazaki C, Noda A, Koike Y, Yamada S, Murohara T, Ozaki N. Association of insomnia and short sleep duration with atherosclerosis risk in the elderly. American journal of hypertension. 2012;25(11):1149-55.
18. Lennon LT, Ramsay SE, Papacosta O, Shaper AG, Wannamethee SG, Whincup PH. Cohort Profile Update: The British Regional Heart Study 1978-2014: 35 years follow-up

For peer review only - http://bmjopen.bmj.com/site/about/guidelines.xhtml

of cardiovascular disease and ageing. International journal of epidemiology. 2015;44(3):826-g.
19. Ramsay SE, Arianayagam DS, Whincup PH, Lennon LT, Cryer J, Papacosta AO, et al. Cardiovascular risk profile and frailty in a population-based study of older British men. Heart (British Cardiac Society). 2015;101(8):616-22.
20. Wannamethee SG, Lowe GD, Whincup PH, Rumley A, Walker M, Lennon L. Physical activity and hemostatic and inflammatory variables in elderly men. Circulation. 2002;105(15):1785-90.
21. Wannamethee SG, Whincup PH, Shaper AG, Rumley A, Lennon L, Lowe GDO. Circulating inflammatory and hemostatic biomarkers are associated with risk of myocardial infarction and coronary death, but not angina pectoris, in older men. Journal of Thrombosis and Haemostasis. 2009;7(10):1605-11.
22. Wannamethee GS, Welsh P, Whincup PH, Lennon L, Papacosta O, Sattar N. N-terminal pro brain natriuretic peptide but not copeptin improves prediction of heart failure over other routine clinical risk parameters in older men with and without cardiovascular disease: population-based study. European Journal of Heart Failure. 2014;16(1):25-32.
23. National Institute for Clinical Excellence. Chronic heart failure: the management of adults with chronic heart failure in primary and secondary care (partial update). (Clinical guideline 108) 2010 [Available from: http://www.nice.org.uk/CG108.
24. Dijk JM, Algra A, van der Graaf Y, Grobbee DE, Bots ML. Carotid stiffness and the risk of new vascular events in patients with manifest cardiovascular disease. The SMART study. European heart journal. 2005;26(12):1213-20.
25. Leng Y, Ahmadi-Abhari S, Wainwright NWJ, Cappuccio FP, Surtees PG, Luben R, et al. Daytime napping, sleep duration and serum C reactive protein: a population-based cohort study. BMJ Open. 2014;4(11):e006071.
26. Kociol RD, Pang PS, Gheorghiade M, Fonarow GC, O'Connor CM, Felker GM. Troponin Elevation in Heart FailurePrevalence, Mechanisms, and Clinical Implications. Journal of the American College of Cardiology. 2010;56(14):1071-8.
27. Kragelund C, Grønning B, Køber L, Hildebrandt P, Steffensen R. N-Terminal Pro–B-Type Natriuretic Peptide and Long-Term Mortality in Stable Coronary Heart Disease. New England Journal of Medicine. 2005;352(7):666-75.
28. Sato Y, Fujiwara H, Takatsu Y. Biochemical markers in heart failure. Journal of cardiology. 2012;59(1):1-7.
29. Laugsand LE, Strand LB, Platou C, Vatten LJ, Janszky I. Insomnia and the risk of incident heart failure: a population study. European heart journal. 2014;35(21):1382-93.
30. Ingelsson E, Lind L, Ärnlöv J, Sundström J. Sleep disturbances independently predict heart failure in overweight middle-aged men. European Journal of Heart Failure. 2007;9(2):184-90.

For peer review only - http://bmjopen.bmj.com/site/about/guidelines.xhtml

STROBE Statement—checklist of items that should be included in reports of observational studies

	Item No	Recommendation
**Title and abstract**	1	(*a*) Indicate the study's design with a commonly used term in the title or the abstract – **PAGE 1**
		(*b*) Provide in the abstract an informative and balanced summary of what was done and what was found – **PAGE 2**
**Introduction**		
Background/rationale	2	Explain the scientific background and rationale for the investigation being reported – **PAGE 4**
Objectives	3	State specific objectives, including any prespecified hypotheses - **PAGE 4**
**Methods**		
Study design	4	Present key elements of study design early in the paper - **PAGE 5-7**
Setting	5	Describe the setting, locations, and relevant dates, including periods of recruitment, exposure, follow-up, and data collection - **PAGE 5-7**
Participants	6	(*a*) *Cohort study*—Give the eligibility criteria, and the sources and methods of selection of participants. Describe methods of follow-up – **PAGE 5**<br>*Case-control study*—Give the eligibility criteria, and the sources and methods of case ascertainment and control selection. Give the rationale for the choice of cases and controls<br>*Cross-sectional study*—Give the eligibility criteria, and the sources and methods of selection of participants
		(*b*) *Cohort study*—For matched studies, give matching criteria and number of exposed and unexposed – **N/A**<br>*Case-control study*—For matched studies, give matching criteria and the number of controls per case
Variables	7	Clearly define all outcomes, exposures, predictors, potential confounders, and effect modifiers. Give diagnostic criteria, if applicable – **PAGE 5-7**
Data sources/ measurement	8*	For each variable of interest, give sources of data and details of methods of assessment (measurement). Describe comparability of assessment methods if there is more than one group – **PAGE 5-7**
Bias	9	Describe any efforts to address potential sources of bias – **PAGE 13**
Study size	10	Explain how the study size was arrived at - **PAGE 5**
Quantitative variables	11	Explain how quantitative variables were handled in the analyses. If applicable, describe which groupings were chosen and why – **PAGE 5-7**
Statistical methods	12	(*a*) Describe all statistical methods, including those used to control for confounding - **PAGE 7**
		(*b*) Describe any methods used to examine subgroups and interactions – **PAGE 7**
		(*c*) Explain how missing data were addressed – **PAGE 5-6**
		(*d*) *Cohort study*—If applicable, explain how loss to follow-up was addressed – **N/A**<br>*Case-control study*—If applicable, explain how matching of cases and controls was addressed<br>*Cross-sectional study*—If applicable, describe analytical methods taking account of sampling strategy
		(*e*) Describe any sensitivity analyses – **N/A**

Continued on next page

For peer review only - http://bmjopen.bmj.com/site/about/guidelines.xhtml

**Results**		
Participants	13*	(a) Report numbers of individuals at each stage of study—eg numbers potentially eligible, examined for eligibility, confirmed eligible, included in the study, completing follow-up, and analysed – **PAGE 5-6**
		(b) Give reasons for non-participation at each stage – **PAGE 5**
		(c) Consider use of a flow diagram – **N/A**
Descriptive data	14*	(a) Give characteristics of study participants (eg demographic, clinical, social) and information on exposures and potential confounders – **PAGE 7-8, 15**
		(b) Indicate number of participants with missing data for each variable of interest – **PAGE 15-19**
		(c) *Cohort study*—Summarise follow-up time (eg, average and total amount) – **PAGE 5**
Outcome data	15*	*Cohort study*—Report numbers of outcome events or summary measures over time – **PAGE 15-19**
		Case-control study—Report numbers in each exposure category, or summary measures of exposure
		Cross-sectional study—Report numbers of outcome events or summary measures
Main results	16	(*a*) Give unadjusted estimates and, if applicable, confounder-adjusted estimates and their precision (eg, 95% confidence interval). Make clear which confounders were adjusted for and why they were included – **PAGE 15-19**
		(*b*) Report category boundaries when continuous variables were categorized – **N/A**
		(*c*) If relevant, consider translating estimates of relative risk into absolute risk for a meaningful time period – **N/A**
Other analyses	17	Report other analyses done—eg analyses of subgroups and interactions, and sensitivity analyses – **PAGE 9-10**
**Discussion**		
Key results	18	Summarise key results with reference to study objectives – **PAGE 7-10**
Limitations	19	Discuss limitations of the study, taking into account sources of potential bias or imprecision. Discuss both direction and magnitude of any potential bias – **PAGE 13**
Interpretation	20	Give a cautious overall interpretation of results considering objectives, limitations, multiplicity of analyses, results from similar studies, and other relevant evidence – **PAGE 11-13**
Generalisability	21	Discuss the generalisability (external validity) of the study results – **PAGE 13**
**Other information**		
Funding	22	Give the source of funding and the role of the funders for the present study and, if applicable, for the original study on which the present article is based – **PAGE 14**

*Give information separately for cases and controls in case-control studies and, if applicable, for exposed and unexposed groups in cohort and cross-sectional studies.

Note: An Explanation and Elaboration article discusses each checklist item and gives methodological background and published examples of transparent reporting. The STROBE checklist is best used in conjunction with this article (freely available on the Web sites of PLoS Medicine at http://www.plosmedicine.org/, Annals of Internal Medicine at http://www.annals.org/, and Epidemiology at http://www.epidem.com/). Information on the STROBE Initiative is available at www.strobe-statement.org.

For peer review only

Self-reported sleep duration and napping, cardiac risk factors and markers of subclinical vascular disease: cross-sectional study in older men

Journal:	*BMJ Open*
Manuscript ID	bmjopen-2017-016396.R1
Article Type:	Research
Date Submitted by the Author:	27-Apr-2017
Complete List of Authors:	Zonoozi, Shahrzad; UCL, Department of Primary Care & Population Health<br>Ramsay, Sheena; Newcastle University, Institute of Health & Society<br>Papacosta, Olia; University College London,<br>Lennon, Lucy; UCL, Department of Primary Care and Population Health<br>Ellins, Elizabeth; Swansea University, Institute of Life Sciences<br>Halcox, Julian; Swansea University, Department of Cardiology<br>Whincup, Peter; St George's University of London,<br>Wannamethee, Goya; UCL, Primary Care and Population Health
**Primary Subject Heading**:	Epidemiology
Secondary Subject Heading:	Cardiovascular medicine
Keywords:	Sleep patterns, Cardiac function, Subclinical atherosclerosis

SCHOLARONE™
Manuscripts

Self-reported sleep duration and napping, cardiac risk factors and markers of subclinical vascular disease: cross-sectional study in older men

Shahrzad Zonoozi[1]; Sheena E Ramsay[2]; Olia Papacosta[1]; Lucy Lennon[1]; Elizabeth A Ellins[3]; Julian P J Halcox[3]; Peter H Whincup[4]; S Goya Wannamethee[1]

[1] UCL Department of Primary Care & Population Health, UCL Medical School, Rowland Hill Street, London, NW3 2PF, UK

[2] Institute of Health & Society, Newcastle University, Richardson Road, Newcastle upon Tyne, NE2 4AX, UK

[3] Institute of Life Sciences, Swansea University, Singleton Park, Swansea, SA2 8PP, UK

[4] Population Health Research Institute, St George's University of London, Cranmer Terrace, London, SW17 0RE, UK

Corresponding author:

Shahrzad Zonoozi, UCL Department of Primary Care & Population Health, UCL Medical School, Rowland Hill Street, London, NW3 2PF, UK. Email: shahrzadz@gmail.com

Word count: 3595; 4 tables

Keywords: sleep patterns, cardiac function, subclinical atherosclerosis

For peer review only - http://bmjopen.bmj.com/site/about/guidelines.xhtml

Abstract

Study Objectives: Daytime sleep has been associated with increased risk of cardiovascular disease and heart failure (HF) but the mechanisms remain unclear. We have investigated the association between daytime and night-time sleep patterns and cardiovascular risk markers in older adults including cardiac markers and subclinical markers of atherosclerosis [arterial stiffness and carotid intima-media thickness (CIMT)].

Methods: Cross sectional study of 1722 surviving men aged 71-92 examined in 2010-2012 across 24 British towns, from a prospective study initiated in 1978-1980. Participants completed a questionnaire and were invited for a physical examination. Men with a history of heart attack or HF (n = 251) were excluded from the analysis.

Results: Self-reported daytime sleep duration was associated with higher fasting glucose and insulin levels (p = 0.02 and 0.01 respectively) even after adjustment for age, BMI, physical activity and social class. Compared to those with no daytime sleep, men with daytime sleep >1 hour, defined as excessive daytime sleepiness (EDS), had a higher risk of raised N-terminal pro brain natriuretic peptide (NT-pro BNP) of ≥400pg/ml, the diagnostic threshold for HF [OR (95% CI) = 1.88 (1.15, 3.1)], higher mean troponin (cTnT), reduced lung function (forced expiratory volume in 1s) and elevated von Willebrand factor (vWF), a marker of endothelial dysfunction. However, EDS was unrelated to CIMT and arterial stiffness. By contrast night-time sleep was only associated with HbA1c (short or long sleep) and arterial stiffness (short sleep).

Conclusions: Daytime sleep duration of >1 hour may be an early indicator of HF.

Word count: 248

For peer review only - http://bmjopen.bmj.com/site/about/guidelines.xhtml

Article Summary

Strength and limitations of this study

- To the best of our knowledge, this is the first study to look at he association between excessive daytime sleepiness (EDS), cardiac markers and vascular parameters.
- Owing to a lack of data, we do not know if the associations seen are true for women, middle aged populations and ethnic minorities.
- As we do not have polysomnography data, we were unable to fully exclude obstructive sleep apnoea as a contributing factor to our findings.
- Our results are based on a cross-sectional analysis and therefore causality cannot be established.

For peer review only

For peer review only - http://bmjopen.bmj.com/site/about/guidelines.xhtml

Introduction

Sleep disturbance is a common complaint, especially amongst older adults. (1) It is increasingly recognised that there is a U shaped association between sleep duration and risk of cardiovascular disease (CVD) and metabolic disturbances – i.e. both short and long sleep durations are associated with an increased risk. (2-4) More recently, studies have shown excessive daytime sleepiness (EDS), one of the more common sleep disturbances reported in the older population, to be associated with increased risks of CVD (5, 6), heart failure (HF) (6, 7) and all-cause mortality. (8-10)

The mechanism of the association between EDS and CVD risk remains unclear. Whilst some studies have looked at the association between EDS and metabolic risk markers (11, 12), few have examined mechanisms by which EDS may influence CVD and in particular incident HF. (6) In particular, the association between EDS and markers of arterial disease and surrogate markers of cardiac function, including NT-pro brain natriuretic peptide (BNP), a marker of ventricular stress, and cardiac troponin T (cTnT), a marker of myocardial injury, have been less studied. Sub-clinical arterial disease markers are increasingly being used as early predictors for atherosclerosis and CVD. A number of non-invasive assessments have been developed, including measures of atherosclerosis such as carotid intima-media thickness (CIMT) and indirect measures of arterial stiffness including carotid distensibility and pulse wave velocity (PWV). Although a number of studies have examined the association between night-time sleep duration and arterial stiffness, (13-17) studies on the association between EDS and vascular risk markers are limited. (5) We have therefore examined the association between daytime sleep as well as night-time sleep with vascular risk factors including metabolic risk factors, inflammatory and endothelial markers, cardiac markers and measures of sub-clinical arterial disease (including arterial stiffness and CIMT).

For peer review only - http://bmjopen.bmj.com/site/about/guidelines.xhtml

Methods

The British Regional Heart Study is a prospective study which recruited a socioeconomically and geographically representative cohort of 7735 men from 24 British towns between 1978 and 1980. The present investigation is based on a reassessment in 2010-2012, when all surviving men (n = 3137), aged between 71–92 years, were sent a postal questionnaire and invited for a 30th year re-examination. 2137 (68%) men completed the postal questionnaire and 1722 (55%) men attended the re-examination. (18) Blood samples were collected after fasting for a minimum of six hours and were stored at −70°C. Ethical approval for data collection was obtained from the relevant research ethics committee. The men were asked if a doctor had ever told them they had a heart attack or HF and these men were excluded from the analysis (n = 251). Of the men with no history of heart attack or HF, 1471 men had physical, blood and ultrasound measurements carried out. This present study is a cross-sectional analysis of the data from the 2010-2012 questionnaires and re-examination.

CVD risk factors: Physical examination included blood pressure (BP), lung function as well as height and weight, from which body mass index (BMI) was calculated. (19) Height standardised forced expiratory volume in 1 second (FEV1) was measured using the Vitalograph Compact II. Details of measurement and classification for smoking status and physical activity in this cohort have been previously described. (20) Details of the men's medication history, including use of antihypertensive, hypnotic and anxiolytic medication, were recorded at the examination and participants were also asked to report symptoms of breathlessness. Measurements of metabolic, inflammatory and endothelial markers were taken as described previously. (19, 21, 22) NT-pro BNP and cTnT were measured using the Elecsys 2010 electrochemiluminescence method (Roche Diagnostics, Burgess Hill, UK). The lowest detectable value of cTnT was 5 and for anyone with a recorded reading of < 5, we used half of the lowest detectable value (i.e. 2.5). NT-pro BNP ≥400pg/ml was defined as high, as per current guidelines issued by the National Institute of Clinical Excellence (NICE)

For peer review only - http://bmjopen.bmj.com/site/about/guidelines.xhtml

for the diagnosis of HF. (23) Prevalent diabetes included men with doctor-diagnosed diabetes and men with fasting blood glucose ≥7 mmol/L. 1402 men had at least one blood measurement (69 missing). Chronic kidney disease (CKD) was defined as estimated glomerular filtration rate of less than 60 using the CKD Epidemiology Collaboration (CKD-EPI) creatinine equation. (24)

Sleep parameters: Reported duration of night sleep was based on response to: “On average, how many hours of sleep do you have each night?” Duration of day sleep was based on response to “On average, how much sleep (if any) do you have during the daytime?” If a response was entered for the night sleep question but no response was indicated for the day sleep question, the answer was set to 0. 1436 men completed the questions on night-time and daytime sleep duration (35 missing). Night-time sleep was categorised into three groups: <6, 6-8.9 and ≥9 hours. Daytime sleep was categorised into four groups: 0 hours, <1 hour, 1 hour and >1 hour. Those sleeping >1 hour during the daytime were defined as having EDS.

Non-invasive markers of arterial disease: Measurements were measured by two vascular technicians in series. Left and right carotid arteries were images using a Z.One Ultra ultrasound system (Zonare Medical Systems, Mountain View, CA) with a 5-10-mHz linear probe. A cross sectional sweep from the base of the common carotid artery to the jaw bone and longitudinal images of the common carotid artery approximately 1cm proximal to the carotid bifurcation were recorded. Peak systolic and end-diastolic common carotid artery diameter and CIMT, the distance between the leading edge of the intima and the media-adventitia interface, were measured using Carotid Analyser software (Medical Imaging Applications, Iowa City, IA). From the longitudinal images, a region of interest (5-10 mm) was selected in a plaque free area, at least 1cm from the bifurcation.

CIMT was measured from three end-diastolic images on each side and a mean of these measures was calculated. Maximum and minimum carotid artery diameter was assessed from three consecutive waveforms and mean distension was calculated as: maximum

For peer review only - http://bmjopen.bmj.com/site/about/guidelines.xhtml

diameter – minimum diameter. The distensibility coefficient was then calculated as described by Dijk *et al.*: distensibility coefficient = [(2 x mean distension/baseline diameter)/mean pulse pressure (kPa)]*1000. (25) Carotid to femoral PWV was assessed using a Vicorder (Skidmore Medical, Bristol UK), with participants in a semi-supine position with their torso at approximately 30˚. A 2x9cm cuff was positioned around the neck with the bladder over the right carotid pulse, and a Hokanson SC10 cuff around the middle of the right thigh. Path length was measured from the sternal notch to the centre of the thigh cuff. The cuffs were simultaneously inflated and traces with a minimum of 3 good quality waveforms recorded. Two PWV measurements, within ≤0.5m/s of each other, were accepted and averaged. 1464 men had at least one non-invasive vascular measurement carried out (7 missing).

Statistical analysis: Distributions of HbA1c, glucose, insulin, CRP, IL-6, NT-pro BNP and cTnT were highly skewed and log transformation was used. Comparisons of baseline characteristics between the sleep groups were carried out using the chi squared test for categorical variables and analysis of variance for continuous variables. Logistic regression models were used to assess the differences in outcomes between categorical groups. In multivariate analyses, age, BMI, physical activity and social class were fitted as continuous variables whilst prevalent diabetes and use of antihypertensive medications was defined as categorical variables. The base groups for the statistical analysis were the group sleeping 6-8.9 hours in the night-time sleep group. A threshold effect was seen in those sleeping >1 hour in the daytime and therefore we compared those sleeping >1 hour with those sleeping ≤1. We also carried out statistical analysis of the trend in the daytime sleep group. All analyses were performed using SAS version 9.3 (SAS, Cary, North Carolina).

Results

The average duration of night-time sleep in the men who responded was 6.8 hours (± 1.4 hours). 15% of the men reported night-time sleep duration of less than 6 hours, while 7% reported sleeping ≥9 hours during the night. 47% of men reported no daytime sleep whilst

For peer review only - http://bmjopen.bmj.com/site/about/guidelines.xhtml

9% reported EDS. Night-time sleep duration was significantly associated with EDS (p = 0.004), with a smaller proportion of those sleeping ≥9 hours reporting EDS. Daytime sleep duration was significantly associated with night-time sleep of less than 6 hours (p = 0.02).

Baseline characteristics

Table 1 describes the baseline characteristics of the study population according to night-time and daytime sleep patterns. A U-shaped relationship was seen between night-time sleep and CVD risk factors. Both short and long night-time sleep were associated with increased age, increased prevalence of physical inactivity, manual workers and breathlessness compared to those with 6-8.9 hours sleep. There was a significant association between night-time sleep and use of hypnotic or anxiolytic medications. Daytime sleep is strongly associated with older age and with many adverse characteristics including physical inactivity, obesity, prevalent diabetes, breathlessness, use of anti-hypertensive medication, depression and CKD. The prevalence of these adverse factors tended to increase with increasing duration of daytime sleep. Daytime sleep was not associated with use of hypnotic or anxiolytic medications.

Night-time sleep and and cardiovascular risk markers

There was a U-shaped association between night-time sleep and metabolic risk factors including HbA1c and glucose (Table 2). The association between night-time sleep and HbA1c remained significant following adjustment for daytime sleep (p = 0.04). In age-adjusted analysis, night-time sleep was significantly associated with IL-6, a marker of inflammation, but this association was attenuated following adjustment for BMI, social class and physical activity (p = 0.05). No significant association was seen between night-time sleep and von Willebrand factor (vWF), a marker of endothelial function, FEV1 (lung function) or cardiac markers (NT-proBNP and cTnT).

For peer review only - http://bmjopen.bmj.com/site/about/guidelines.xhtml

Daytime sleep and cardiovascular risk markers

In age-adjusted analysis, metabolic risk factors including HDL-C, triglycerides, HbA1c and insulin tended to increase with increasing hours of daytime sleep (Table 3). No association was seen with BP. The increasing trend was attenuated after adjustment for age, BMI, social class and physical activity. However compared to those who slept $\leq$1 hour, those with EDS (>1 hour) showed significantly higher mean levels of HbA1c, glucose and insulin (Table 3). Mean levels of glucose and insulin remained significantly higher in those with EDS ($p = 0.03$ and 0.01 respectively) even after further adjustment for prevalent diabetes and night-time sleep.

CRP increased with increasing daytime sleep duration, even after full adjustment (p trend = 0.04). Lung function decreased with increasing daytime sleep duration with levels markedly reduced in those with EDS compared to those reporting $\leq$1 hour daytime sleep ($p < 0.0001$). Only EDS was associated with significantly higher mean vWF levels ($p = 0.03$). When we examined the odds of having high vWF (top quintile) and low FEV1 (lowest quintile), those with EDS were 1.8 times more likely to have a high vWF [OR (95% CI) = 1.82 (1.17, 2.83)] and 2.4 times more likely to have a low FEV1 [OR (95% CI) = 2.4 (1.53, 3.74)] compared to those who reported no daytime sleep.

Mean levels of NT-pro BNP and cTnT (cardiac markers) were significantly higher only in those with EDS following full adjustment ($p = 0.02$ and 0.03 respectively). This association remained significant even after adjustment for CKD. Men with EDS were more likely to have high (top quintile) mean NT-pro BNP [OR (95% CI) = 1.69 (1.06, 2.7)] and cTnT [OR (95% CI) = 1.75 (1.09, 2.8)] compared to those who reported no daytime sleep. However, this was attenuated following adjustment for prevalent diabetes, use of antihypertensive medication and depression [OR (95% CI) = 1.59 (0.99, 2.55)] and [OR (95% CI) = 1.62 (1.00, 2.62)] respectively, although it remained significant for cTnT. We further looked at men with a NT-pro BNP of $\geq$400pg/ml, the diagnostic threshold for HF. Men who reported EDS had a higher

For peer review only - http://bmjopen.bmj.com/site/about/guidelines.xhtml

odds of having an NT-pro BNP of ≥400pg/ml [OR (95% CI) = 1.88 (1.15, 3.1)]. This association remained following adjustment for prevalent diabetes, CKD, use of antihypertensive medication and depression [OR (95% CI) = 1.72 (1.03, 2.87)]. Exclusion of men using anxiolytic and hypnotic medications did not significantly alter this [OR (95% CI) = 1.76 (1.05, 2.95)].

Sleep patterns and non-invasive markers of arterial disease

Table 4 shows the associations between sleeping patterns and non-invasive vascular measurements. There was a significant association between those sleeping less than 6 hours and mean PWV and distensibility when compared to those sleeping 6-8.9 hours in the fully adjusted model (p = 0.04 and 0.03 respectively). Men who slept less than 6 hours during the night-time were 1.6 times more likely to have a low (lowest quintile) mean distensibility compared to those who slept 6-8.9 hours in the fully adjusted model [OR (95% CI) = 1.56 (1.09, 2.23)]. There was no significant association between daytime sleep and markers of arterial stiffness. There was no association between sleeping patterns and CIMT in the study population.

For peer review only - http://bmjopen.bmj.com/site/about/guidelines.xhtml

Discussion

We have confirmed previous reports of an association between night-time sleep duration (4) and daytime sleep duration (11, 12) with metabolic risk factors. Our findings extend previous studies by investigating the association between EDS and a wide range of cardiac markers and vascular measures not previously examined. Our study has shown that in a representative sample of British men aged 71–92 years with no prior history of heart attack or HF, those who sleep more than one hour during the day have a number of adverse characteristics including higher mean levels of cardiac markers, metabolic risk factors, inflammatory and endothelial markers and reduced lung function. These findings may point to pathways for the association observed between EDS and CVD and all-cause mortality. (8)

EDS and metabolic and cardiac risk markers

EDS, defined in our study as daytime sleep of >1 hour, is associated with many adverse characteristics such as obesity, physical inactivity, diabetes, breathlessness, use of antihypertensive therapy and with an adverse pattern of metabolic risk factors including HbA1c, glucose and insulin. Our results confirm previous cross-sectional analyses which have shown EDS to be associated with the metabolic syndrome and longer habitual napping duration to be associated with an increased risk of impaired fasting glucose and diabetes mellitus. (11, 12) There was a significant linear relationship between increasing daytime sleep and mean CRP levels, with those reporting EDS having higher levels of mean CRP. This confirms results from a previous study which showed self-reported daytime napping is associated with higher levels of CRP in older British adults. (26) The association seen between daytime sleep and these markers of inflammation may provide an explanation for the excess all-cause mortality observed in those with daytime napping or EDS. (8, 9) Whilst both of these studies have adjusted for diabetes, neither has adjusted for inflammation.

We found a significant association between EDS and high levels of NT-pro BNP and cTnT, both of which are biomarkers of HF. (27, 28) NT-pro BNP is produced in response to cardiac

For peer review only - http://bmjopen.bmj.com/site/about/guidelines.xhtml

wall stress and is a strong predictor of HF. (22) Troponin T is a plasma marker of myocyte necrosis, which has been positively associated with incident CVD, HF and all-cause mortality. (29) Most notably men who reported EDS had a higher odds of having NT-pro BNP of ≥400pg/ml (the diagnostic threshold for HF). As far as we are aware, this is the first study to look at the association between EDS and these cardiac markers.

A strong association was also seen between EDS and elevated vWF (marker of endothelial dysfunction) and low FEV1, markers associated with HF. These findings suggest that EDS may be an early indicator of HF. This supports a recent report from our study when the men were aged 63-82 years which showed EDS to be associated with the risk of developing overt HF during 9 year follow-up. (7) In that analyses cardiac markers and lung function were not available.

The association between EDS and HF may be explained by underlying respiratory illness such as obstructive sleep apnoea (OSA). Indeed within our cohort, EDS was significantly associated with obesity, self-reported breathlessness and reduced lung function. EDS may be a result of disturbed night-time sleep and difficulty falling asleep, both of which have been associated with HF. (30, 31) However, the associations we observed with cardiac markers and lung function were not seen in men with a short or long duration of night-time sleep and further analyses showed no association between these markers and those reporting difficulty falling asleep.

Non-invasive markers of arterial disease

In contrast to night-time sleep we did not observe any significant association between daytime sleep and markers of arterial stiffness. Short night-time sleep but not long night-time sleep was associated with arterial stiffness (PWV and carotid distensibility), when compared to those sleeping 6-8.9 hours. There was no association between sleeping patterns and CIMT in the study population. This is in contrast to previous studies which have shown an

For peer review only - http://bmjopen.bmj.com/site/about/guidelines.xhtml

association between short night-time sleep (17) and EDS (5) with CIMT. The differences observed may be due to use of brachial-ankle PWV (13, 15), rather than carotid-femoral PWV, different classification of long night-time sleep (13, 14) and a younger study population. (15)

Strengths and limitations

The strengths of our study include the fact that it is a study of older men, a group who are at high risk of vascular disease and the wide range of vascular parameters which we have looked into. Limitations of our study include the lack of representation of women, middle aged populations and ethnic minority groups which limits generalisability. Our data is based on self-reported sleeping patterns at a single time point and it is possible that night-time and daytime sleep duration have been misclassified. Our analysis of night-time sleep relies on average duration of night-time sleep and we do not have measures of interruption or total time spent in bed. As we do not have polysomnography data, we were unable to fully exclude OSA as a contributing factor to our findings. Data regarding occupation was not available and we were unable to assess if previous shift work, which has been reported to be associated with cardiovascular and metabolic diseases, is related to the adverse cardiovascular profile we noted in those with EDS. We appreciate that our results are based on a cross-sectional analysis and therefore causality cannot be established and the direction of the effect is unknown. It is also possible that there is an element of survival bias in our data; it may be that men who attended the re-examination were healthier and this may account for the null associations seen.

Conclusions

EDS, defined as daytime sleep of more than one hour, is associated with an adverse cardiovascular profile and may be an early indicator of HF. Clinically, this is important in the

For peer review only - http://bmjopen.bmj.com/site/about/guidelines.xhtml

assessment of older patients who seek medical advice for EDS, in whom indicators of HF should be sought and further investigations carried out if necessary.

Disclosures

The authors report no conflicts of interest.

Funding

The British Regional Heart Study is a British Heart Foundation (BHF) research group. This work was supported by a British Heart Foundation programme grant (RG/13/16/30528) and project grant (PG/09/024, PG/13/41/30304).

Contributions

SGW initiated the concept and design of the paper. SZ analysed the data and drafted the manuscript. SGW, JPJH, EAE, PHW contributed to the interpretation of data. OP contributed to the analysis of the paper. EAE, JPJH, LL, PHW and SGW contributed to the acquisition of the data. All authors revised it critically for important intellectual content and approved the final version of the manuscript.

Data Sharing Statement

Further details of the Study can be found on http://www.ucl.ac.uk/pcph/research-groups-themes/brhs-pub.

For general data sharing enquiries, please contact Lucy Lennon (l.lennon@ucl.ac.uk).

For peer review only - http://bmjopen.bmj.com/site/about/guidelines.xhtml

For peer review only

Table 1 - Baseline characteristics of the study population

		Age mean ±std	Obese (%)	Cigarette Smoker (%)	Inactive (%)	Manual social class (%)	Diabetic (%)	Breathlessness (%)	Using Antihypertensives (%)	Depression (%)	CKD (%)
**Night-time Sleep Duration (hours)**	**< 6 (n = 213[a])**	78.76±4.6	21.53	0.94	48.36	57.35	18.31	40.72	53.99	11.43	29
	**6-8.9 (n = 1126[a])**	78.22±4.6	18.78	3.83	33.66	42.4	14.87	19.32	48.85	8.39	29.36
	**≥ 9 (n = 97[a])**	79.24±4.9	22.11	4.12	38.14	48.45	19.79	25	52.58	11.34	38.71
	**p**	0.05	0.52	0.10	0.0002 *	0.002 *	0.24	<.0001 *	0.33	0.27	0.16
**Daytime Sleep Duration (hours)**	**0 (n = 680[a])**	77.83±4.4	16.1	2.65	30.74	46.1	12.24	17.59	45.88	7.41	26.42
	**<1 (n = 290[a])**	78.04±4.4	15.63	2.76	33.45	40	14.88	21.61	54.14	10.42	30.94
	**1 (n = 336[a])**	78.79±4.7	24.17	5.07	40.77	44.61	20	27.01	49.7	8.98	32.81
	**>1 (n = 130[a])**	80.05±5	33.33	4.62	58.46	51.54	24.62	41.46	61.54	14.62	39.17
	**p**	<.0001 *	<.0001 *	0.18	<.0001 *	0.15	0.0003 *	<.0001 *	0.004 *	0.05	0.02 *

a, maximum n in group, varies slightly with missing co-variate data

CKD, chronic kidney disease

For peer review only - http://bmjopen.bmj.com/site/about/guidelines.xhtml

Table 2 - Night-time sleep duration vs metabolic risk factors, inflammatory and endothelial markers, lung function and cardiac markers.

		Night Sleep (hours)			
		< 6 (n = 213[d])	6-8.9 (n = 1126[d])	≥ 9 (n = 97[d])	p
**Metabolic risk factors [mean (95% CI)]**					
**SBP (mmHg)**	**A**	146.04 (143.52, 148.56)	147.31 (146.21, 148.4)	150.73 (146.99, 154.47)	0.12
**DBP (mmHg)**	**A**	76.59 (75.07, 78.11)	77.46 (76.8, 78.12)	77.25 (74.99, 79.5)	0.59
**HDL-C (mmol/L)**	**A**	1.47 (1.41, 1.53)	1.48 (1.45, 1.5)	1.37 (1.28, 1.46)	0.07
**Triglycerides (mmol/L)**	**A**	1.3 (1.21, 1.39)	1.28 (1.25, 1.32)	1.39 (1.26, 1.52)	0.31
**HbA1c[c] (%)**	**A**	5.93 (5.85, 6.02)	5.75 (5.72, 5.79)	5.88 (5.76, 6)	0.0001 *
	**B**	5.9 (5.82, 5.98)	5.76 (5.73, 5.79)	5.86 (5.74, 5.97)	0.003 *
**Glucose[c] (mmol/L)**	**A**	5.78 (5.62, 5.94)	5.55 (5.48, 5.61)	5.72 (5.49, 5.95)	0.02 *
	**B**	5.74 (5.58, 5.9)	5.56 (5.49, 5.62)	5.68 (5.45, 5.91)	0.08
**Insulin[c] (mU/L)**	**A**	8.26 (7.54, 9.04)	7.92 (7.62, 8.24)	7.82 (6.84, 8.94)	0.69
**Inflammatory and endothelial markers [mean (95% CI)]**					
**CRP[c] (mg/L)**	**A**	1.51 (1.28, 1.78)	1.34 (1.25, 1.44)	1.46 (1.15, 1.87)	0.37
**IL-6[c] (pg/mL)**	**A**	3.03 (2.74, 3.35)	2.98 (2.86, 3.11)	3.72 (3.21, 4.3)	0.02 *
	**B**	2.94 (2.67, 3.25)	3 (2.87, 3.13)	3.61 (3.12, 4.18)	0.05
**vWF (IU/dL)**	**A**	140.97 (130.97, 150.97)	133.27 (128.91, 137.63)	119.04 (104.2, 133.89)	0.06
**Lung function [mean (95% CI)]**					
**FEV1 (L)**	**A**	2.43 (2.35, 2.5)	2.46 (2.42, 2.49)	2.44 (2.32, 2.55)	0.76
**Cardiac markers [mean (95% CI)]**					
**NT-pro BNP[c] (pg/mL)**	**A**	121.67 (101.33, 146.1)	126.45 (116.87, 136.81)	134.61 (102.8, 176.25)	0.83
**cTnT[c] (pg/ml)**	**A**	11.25 (10.29, 12.3)	10.82 (10.41, 11.24)	10.14 (8.89, 11.57)	0.44

A, adjusted for age

B, adjusted for age, BMI, social class and physical activity

c, geometeric mean

d, maximum n in group, varies slightly with missing co-variate data

SBP, systolic blood pressure; DBP, diastolic blood pressure; HDL-C, high density lipoprotein cholesterol; HbA1c, glycated haemoglobin; CRP, C-reactive protein; IL-6, interleukin 6; vWF, von Willebrand factor; FEV1, forced expiratory volume in 1s; NT-pro BNP, N-terminal pro brain natriuretic peptide; cTnT, cardiac troponin T

For peer review only

For peer review only - http://bmjopen.bmj.com/site/about/guidelines.xhtml

Table 3 – Daytime sleep duration vs metabolic risk factors, inflammatory and endothelial markers, lung function and cardiac markers.

		Daytime Sleep Duration (Hours) 0 (n = 680[d])	<1 (n = 290[d])	1 (n = 336[d])	>1 (n = 130[d])	p value trend	p value >1h vs others
**Metabolic risk factors [mean (95% CI)]**							
**SBP (mmHg)**	**A**	147.46 (146.05, 148.88)	148.76 (146.59, 150.92)	147.14 (145.13, 149.15)	144.13 (140.88, 147.39)	0.90	0.04 *
	**B**	147.49 (146.07, 148.9)	148.59 (146.42, 150.75)	146.97 (144.95, 148.99)	144.78 (141.44, 148.11)	0.96	0.12
**DBP (mmHg)**	**A**	77.41 (76.56, 78.26)	78.08 (76.77, 79.38)	77.16 (75.95, 78.37)	75.54 (73.57, 77.5)	0.98	0.06
	**B**	77.56 (76.7, 78.41)	78.04 (76.74, 79.35)	76.94 (75.73, 78.16)	75.64 (73.63, 77.65)	0.74	0.09
**HDL-C (mmol/L)**	**A**	1.5 (1.47, 1.54)	1.47 (1.42, 1.52)	1.44 (1.39, 1.49)	1.33 (1.25, 1.41)	0.01 *	0.0002 *
	**B**	1.49 (1.46, 1.52)	1.46 (1.41, 1.5)	1.46 (1.42, 1.51)	1.41 (1.33, 1.48)	0.14	0.10
**Triglycerides (mmol/L)**	**A**	1.24 (1.19, 1.29)	1.3 (1.23, 1.38)	1.35 (1.28, 1.42)	1.42 (1.31, 1.54)	0.01 *	0.02 *
	**B**	1.26 (1.21, 1.3)	1.33 (1.26, 1.4)	1.33 (1.26, 1.4)	1.34 (1.22, 1.45)	0.03 *	0.47
**HbA1c[c] (%)**	**A**	5.73 (5.69, 5.78)	5.74 (5.68, 5.81)	5.87 (5.8, 5.93)	5.99 (5.88, 6.1)	0.01 *	0.0003 *
	**B**	5.75 (5.7, 5.79)	5.77 (5.7, 5.83)	5.84 (5.78, 5.91)	5.9 (5.79, 6.01)	0.04 *	0.04 *
**Glucose[c] (mmol/L)**	**A**	5.52 (5.43, 5.6)	5.48 (5.36, 5.61)	5.7 (5.58, 5.83)	5.98 (5.77, 6.2)	0.09	0.0002 *
	**B**	5.54 (5.45, 5.62)	5.51 (5.38, 5.63)	5.68 (5.56, 5.8)	5.86 (5.65, 6.07)	0.25	0.01 *
**Insulin[c] (mU/L)**	**A**	7.5 (7.13, 7.88)	7.43 (6.89, 8.02)	8.69 (8.09, 9.33)	10.36 (9.21, 11.65)	0.01 *	<.0001 *
	**B**	7.7 (7.36, 8.07)	7.68 (7.16, 8.24)	8.31 (7.77, 8.88)	9.29 (8.32, 10.37)	0.15	0.005 *
**Inflammatory and endothelial markers [mean (95% CI)]**							
**CRP[c] (mg/L)**	**A**	1.24 (1.13, 1.36)	1.36 (1.19, 1.57)	1.56 (1.37, 1.78)	1.67 (1.35, 2.08)	0.01 *	0.07
	**B**	1.27 (1.16, 1.39)	1.4 (1.22, 1.61)	1.5 (1.32, 1.71)	1.51 (1.21, 1.89)	0.04 *	0.37
**IL-6[c] (pg/mL)**	**A**	2.88 (2.73, 3.05)	3.01 (2.76, 3.28)	3.19 (2.95, 3.45)	3.58 (3.14, 4.09)	0.03 *	0.01 *
	**B**	2.92 (2.76, 3.08)	3.06 (2.81, 3.33)	3.13 (2.89, 3.39)	3.34 (2.92, 3.81)	0.10	0.15
**vWF (IU/dL)**	**A**	132.65 (127.06, 138.24)	127.34 (118.81, 135.87)	134.92 (126.85, 142.98)	148.03 (135.05, 161.01)	1.00	0.02 *
	**B**	132.76 (127.1, 138.41)	127.64 (119.03, 136.24)	134.88 (126.7, 143.07)	147.74 (134.39, 161.1)	0.96	0.03 *
**Lung function [mean (95% CI)]**							
**FEV1 (L)**	**A**	2.52 (2.48, 2.57)	2.46 (2.39, 2.52)	2.37 (2.31, 2.44)	2.21 (2.11, 2.31)	<.0001 *	<.0001 *
	**B**	2.52 (2.48, 2.56)	2.44 (2.38, 2.51)	2.38 (2.32, 2.44)	2.26 (2.15, 2.36)	<.0001 *	<.0001 *
**Cardiac markers [mean (95% CI)]**							
**NT-pro BNP[c] (pg/mL)**	**A**	122.31 (110.53, 135.34)	110.95 (95.15, 129.37)	135.15 (117.08, 156.02)	171.15 (134.5, 217.78)	0.70	0.01 *
	**B**	123.28 (111.4, 136.43)	111.2 (95.39, 129.62)	135.76 (117.52, 156.82)	167.33 (130.92, 213.88)	0.82	0.02 *
**cTnT[c] (pg/ml)**	**A**	10.33 (9.83, 10.85)	10.45 (9.7, 11.27)	11.59 (10.8, 12.43)	12.79 (11.37, 14.38)	0.03 *	0.004 *
	**B**	10.41 (9.91, 10.93)	10.52 (9.77, 11.33)	11.31 (10.55, 12.13)	12.3 (10.93, 13.86)	0.12	0.03 *

For peer review only - http://bmjopen.bmj.com/site/about/guidelines.xhtml

A, adjusted for age

B, adjusted for age, BMI, social class and physical activity

c, geometeric mean

d, maximum n in group, varies slightly with missing co-variate data

SBP, systolic blood pressure; DBP, diastolic blood pressure; HDL-C, high density lipoprotein cholesterol; HbA1c, glycated haemoglobin; CRP, C-reactive protein; IL-6, interleukin 6; vWF, von Willebrand factor; FEV1, forced expiratory volume in 1s; NT-pro BNP, N-terminal pro brain natriuretic peptide; cTnT, cardiac troponin T

For peer review only

For peer review only - http://bmjopen.bmj.com/site/about/guidelines.xhtml

Table 4 – Sleeping patterns vs non-invasive vascular measurements.

		CIMT (mm)		PWV (m/s)		Distensibility ($10^{-3}kPa^{-1}$)	
		Age-adjusted	Additional Adj[a]	Age-adjusted	Additional Adj[a]	Age-adjusted	Additional Adj[a]
		mean (95% CI)	mean (95% CI)	mean (95% CI)	mean (95% CI)	mean (95% CI)	mean (95% CI)
Night-time Sleep Duration (hours)	< 6 (n = 209[b])	0.81 (0.78, 0.83)	0.8 (0.78, 0.82)	10.48 (10.24, 10.71)	10.45 (10.22, 10.69)	11.66 (11.11, 12.21)	11.62 (11.06, 12.18)
	6-8.9 (n = 1125[b])	0.81 (0.8, 0.82)	0.81 (0.8, 0.82)	10.18 (10.08, 10.27)	10.18 (10.08, 10.28)	12.29 (12.06, 12.53)	12.31 (12.08, 12.55)
	≥ 9 (n = 95[b])	0.82 (0.79, 0.85)	0.82 (0.79, 0.85)	10.25 (9.91, 10.6)	10.25 (9.9, 10.59)	11.71 (10.9, 12.51)	11.81 (11, 12.63)
	p	0.67	0.64	0.07	0.11	0.06	0.06
Daytime Sleep Duration (hours)	0 (n = 677[b])	0.81 (0.79, 0.82)	0.81 (0.8, 0.82)	10.25 (10.12, 10.38)	10.25 (10.12, 10.38)	12.23 (11.93, 12.53)	12.2 (11.89, 12.5)
	<1 (n = 290[b])	0.81 (0.79, 0.82)	0.81 (0.79, 0.82)	10.15 (9.96, 10.35)	10.16 (9.96, 10.36)	12.49 (12.03, 12.96)	12.5 (12.04, 12.97)
	1 (n = 334[b])	0.8 (0.79, 0.82)	0.8 (0.78, 0.82)	10.2 (10.01, 10.38)	10.2 (10.01, 10.38)	11.81 (11.38, 12.25)	11.88 (11.44, 12.32)
	>1 (n = 128[b])	0.84 (0.81, 0.87)	0.83 (0.8, 0.86)	10.29 (9.99, 10.6)	10.29 (9.98, 10.6)	11.96 (11.25, 12.67)	12.12 (11.39, 12.85)
	p	0.17	0.37	0.80	0.85	0.18	0.30

a, adjusted for age, BMI, social class and physical activity

b, maximum n in group

CIMT, carotid intima-media thickness; PWV, pulse wave velocity

For peer review only - http://bmjopen.bmj.com/site/about/guidelines.xhtml

Bibliography

1. Flamer HE. Sleep disorders in the elderly. Australian and New Zealand Journal of Medicine. 1996;26(1):96-104.
2. Grandner MA, Sands-Lincoln MR, Pak VM, Garland SN. Sleep duration, cardiovascular disease, and proinflammatory biomarkers. Nature and science of sleep. 2013;5:93-107.
3. Jackson CL, Redline S, Emmons KM. Sleep as a potential fundamental contributor to disparities in cardiovascular health. Annual review of public health. 2015;36:417-40.
4. Knutson KL. Sleep duration and cardiometabolic risk: a review of the epidemiologic evidence. Best practice & research Clinical endocrinology & metabolism. 2010;24(5):731-43.
5. Blachier M, Dauvilliers Y, Jaussent I, Helmer C, Ritchie K, Jouven X, et al. Excessive daytime sleepiness and vascular events: the Three City Study. Annals of neurology. 2012;71(5):661-7.
6. Newman AB, Spiekerman CF, Enright P, Lefkowitz D, Manolio T, Reynolds CF, et al. Daytime sleepiness predicts mortality and cardiovascular disease in older adults. The Cardiovascular Health Study Research Group. Journal of the American Geriatrics Society. 2000;48(2):115-23.
7. Wannamethee SG, Papacosta O, Lennon L, Whincup PH. Self-Reported Sleep Duration, Napping, and Incident Heart Failure: Prospective Associations in the British Regional Heart Study. Journal of the American Geriatrics Society. 2016.
8. Leng Y, Wainwright NW, Cappuccio FP, Surtees PG, Hayat S, Luben R, et al. Daytime napping and the risk of all-cause and cause-specific mortality: a 13-year follow-up of a British population. American journal of epidemiology. 2014;179(9):1115-24.
9. Tanabe N, Iso H, Seki N, Suzuki H, Yatsuya H, Toyoshima H, et al. Daytime napping and mortality, with a special reference to cardiovascular disease: the JACC study. International journal of epidemiology. 2010;39(1):233-43.
10. Zhong G, Wang Y, Tao T, Ying J, Zhao Y. Daytime napping and mortality from all causes, cardiovascular disease, and cancer: a meta-analysis of prospective cohort studies. Sleep medicine. 2015;16(7):811-9.
11. Hayley AC, Williams LJ, Kennedy GA, Berk M, Brennan SL, Pasco JA. Excessive daytime sleepiness and metabolic syndrome: a cross-sectional study. Metabolism. 2015;64(2):244-52.
12. Fang W, Li Z, Wu L, Cao Z, Liang Y, Yang H, et al. Longer habitual afternoon napping is associated with a higher risk for impaired fasting plasma glucose and diabetes mellitus in older adults: results from the Dongfeng–Tongji cohort of retired workers. Sleep medicine. 2013;14(10):950-4.
13. Tsai TC, Wu JS, Yang YC, Huang YH, Lu FH, Chang CJ. Long sleep duration associated with a higher risk of increased arterial stiffness in males. Sleep. 2014;37(8):1315-20.
14. Abe T, Aoki T, Yata S, Okada M. Sleep duration is significantly associated with carotid artery atherosclerosis incidence in a Japanese population. Atherosclerosis. 2011;217(2):509-13.
15. Yoshioka E, Saijo Y, Kita T, Okada E, Satoh H, Kawaharada M, et al. Relation between self-reported sleep duration and arterial stiffness: a cross-sectional study of middle-aged Japanese civil servants. Sleep. 2011;34(12):1681-6.
16. Wolff B, Volzke H, Schwahn C, Robinson D, Kessler C, John U. Relation of self-reported sleep duration with carotid intima-media thickness in a general population sample. Atherosclerosis. 2008;196(2):727-32.
17. Nakazaki C, Noda A, Koike Y, Yamada S, Murohara T, Ozaki N. Association of insomnia and short sleep duration with atherosclerosis risk in the elderly. American journal of hypertension. 2012;25(11):1149-55.
18. Lennon LT, Ramsay SE, Papacosta O, Shaper AG, Wannamethee SG, Whincup PH. Cohort Profile Update: The British Regional Heart Study 1978-2014: 35 years follow-up of cardiovascular disease and ageing. International journal of epidemiology. 2015;44(3):826-g.
19. Ramsay SE, Arianayagam DS, Whincup PH, Lennon LT, Cryer J, Papacosta AO, et al. Cardiovascular risk profile and frailty in a population-based study of older British men. Heart. 2015;101(8):616-22.

For peer review only - http://bmjopen.bmj.com/site/about/guidelines.xhtml

20. Wannamethee SG, Lowe GD, Whincup PH, Rumley A, Walker M, Lennon L. Physical activity and hemostatic and inflammatory variables in elderly men. Circulation. 2002;105(15):1785-90.
21. Wannamethee SG, Whincup PH, Shaper AG, Rumley A, Lennon L, Lowe GDO. Circulating inflammatory and hemostatic biomarkers are associated with risk of myocardial infarction and coronary death, but not angina pectoris, in older men. Journal of Thrombosis and Haemostasis. 2009;7(10):1605-11.
22. Wannamethee GS, Welsh P, Whincup PH, Lennon L, Papacosta O, Sattar N. N-terminal pro brain natriuretic peptide but not copeptin improves prediction of heart failure over other routine clinical risk parameters in older men with and without cardiovascular disease: population-based study. European Journal of Heart Failure. 2014;16(1):25-32.
23. National Institute for Clinical Excellence. Chronic heart failure: the management of adults with chronic heart failure in primary and secondary care (partial update). (Clinical guideline 108) 2010 [Available from: http://www.nice.org.uk/CG108.
24. Levey AS, Stevens LA, Schmid CH, et al. A new equation to estimate glomerular filtration rate. Annals of Internal Medicine. 2009;150(9):604-12.
25. Dijk JM, Algra A, van der Graaf Y, Grobbee DE, Bots ML. Carotid stiffness and the risk of new vascular events in patients with manifest cardiovascular disease. The SMART study. European heart journal. 2005;26(12):1213-20.
26. Leng Y, Ahmadi-Abhari S, Wainwright NWJ, Cappuccio FP, Surtees PG, Luben R, et al. Daytime napping, sleep duration and serum C reactive protein: a population-based cohort study. BMJ Open. 2014;4(11):e006071.
27. Kociol RD, Pang PS, Gheorghiade M, Fonarow GC, O'Connor CM, Felker GM. Troponin Elevation in Heart FailurePrevalence, Mechanisms, and Clinical Implications. Journal of the American College of Cardiology. 2010;56(14):1071-8.
28. Kragelund C, Grønning B, Køber L, Hildebrandt P, Steffensen R. N-Terminal Pro–B-Type Natriuretic Peptide and Long-Term Mortality in Stable Coronary Heart Disease. New England Journal of Medicine. 2005;352(7):666-75.
29. Sato Y, Fujiwara H, Takatsu Y. Biochemical markers in heart failure. Journal of cardiology. 2012;59(1):1-7.
30. Laugsand LE, Strand LB, Platou C, Vatten LJ, Janszky I. Insomnia and the risk of incident heart failure: a population study. European heart journal. 2014;35(21):1382-93.
31. Ingelsson E, Lind L, Ärnlöv J, Sundström J. Sleep disturbances independently predict heart failure in overweight middle-aged men. European Journal of Heart Failure. 2007;9(2):184-90.

For peer review only - http://bmjopen.bmj.com/site/about/guidelines.xhtml

STROBE Statement—checklist of items that should be included in reports of observational studies

	Item No	Recommendation
**Title and abstract**	1	(*a*) Indicate the study's design with a commonly used term in the title or the abstract – **PAGE 1**
		(*b*) Provide in the abstract an informative and balanced summary of what was done and what was found – **PAGE 2**
**Introduction**		
Background/rationale	2	Explain the scientific background and rationale for the investigation being reported – **PAGE 4**
Objectives	3	State specific objectives, including any prespecified hypotheses - **PAGE 4**
**Methods**		
Study design	4	Present key elements of study design early in the paper - **PAGE 5-7**
Setting	5	Describe the setting, locations, and relevant dates, including periods of recruitment, exposure, follow-up, and data collection - **PAGE 5-7**
Participants	6	(*a*) *Cohort study*—Give the eligibility criteria, and the sources and methods of selection of participants. Describe methods of follow-up – **PAGE 5**<br>*Case-control study*—Give the eligibility criteria, and the sources and methods of case ascertainment and control selection. Give the rationale for the choice of cases and controls<br>*Cross-sectional study*—Give the eligibility criteria, and the sources and methods of selection of participants
		(*b*) *Cohort study*—For matched studies, give matching criteria and number of exposed and unexposed – **N/A**<br>*Case-control study*—For matched studies, give matching criteria and the number of controls per case
Variables	7	Clearly define all outcomes, exposures, predictors, potential confounders, and effect modifiers. Give diagnostic criteria, if applicable – **PAGE 5-7**
Data sources/ measurement	8*	For each variable of interest, give sources of data and details of methods of assessment (measurement). Describe comparability of assessment methods if there is more than one group – **PAGE 5-7**
Bias	9	Describe any efforts to address potential sources of bias – **PAGE 13**
Study size	10	Explain how the study size was arrived at - **PAGE 5**
Quantitative variables	11	Explain how quantitative variables were handled in the analyses. If applicable, describe which groupings were chosen and why – **PAGE 5-7**
Statistical methods	12	(*a*) Describe all statistical methods, including those used to control for confounding - **PAGE 7**
		(*b*) Describe any methods used to examine subgroups and interactions – **PAGE 7**
		(*c*) Explain how missing data were addressed – **PAGE 5-6**
		(*d*) *Cohort study*—If applicable, explain how loss to follow-up was addressed – **N/A**<br>*Case-control study*—If applicable, explain how matching of cases and controls was addressed<br>*Cross-sectional study*—If applicable, describe analytical methods taking account of sampling strategy
		(*e*) Describe any sensitivity analyses – **N/A**

Continued on next page

For peer review only - http://bmjopen.bmj.com/site/about/guidelines.xhtml

**Results**		
Participants	13*	(a) Report numbers of individuals at each stage of study—eg numbers potentially eligible, examined for eligibility, confirmed eligible, included in the study, completing follow-up, and analysed – **PAGE 5-6**
		(b) Give reasons for non-participation at each stage – **PAGE 5**
		(c) Consider use of a flow diagram – **N/A**
Descriptive data	14*	(a) Give characteristics of study participants (eg demographic, clinical, social) and information on exposures and potential confounders – **PAGE 7-8, 15**
		(b) Indicate number of participants with missing data for each variable of interest – **PAGE 15-19**
		(c) *Cohort study*—Summarise follow-up time (eg, average and total amount) – **PAGE 5**
Outcome data	15*	*Cohort study*—Report numbers of outcome events or summary measures over time – **PAGE 15-19**
		Case-control study—Report numbers in each exposure category, or summary measures of exposure
		Cross-sectional study—Report numbers of outcome events or summary measures
Main results	16	(*a*) Give unadjusted estimates and, if applicable, confounder-adjusted estimates and their precision (eg, 95% confidence interval). Make clear which confounders were adjusted for and why they were included – **PAGE 15-19**
		(*b*) Report category boundaries when continuous variables were categorized – **N/A**
		(*c*) If relevant, consider translating estimates of relative risk into absolute risk for a meaningful time period – **N/A**
Other analyses	17	Report other analyses done—eg analyses of subgroups and interactions, and sensitivity analyses – **PAGE 9-10**
**Discussion**		
Key results	18	Summarise key results with reference to study objectives – **PAGE 7-10**
Limitations	19	Discuss limitations of the study, taking into account sources of potential bias or imprecision. Discuss both direction and magnitude of any potential bias – **PAGE 13**
Interpretation	20	Give a cautious overall interpretation of results considering objectives, limitations, multiplicity of analyses, results from similar studies, and other relevant evidence – **PAGE 11-13**
Generalisability	21	Discuss the generalisability (external validity) of the study results – **PAGE 13**
**Other information**		
Funding	22	Give the source of funding and the role of the funders for the present study and, if applicable, for the original study on which the present article is based – **PAGE 14**

*Give information separately for cases and controls in case-control studies and, if applicable, for exposed and unexposed groups in cohort and cross-sectional studies.

Note: An Explanation and Elaboration article discusses each checklist item and gives methodological background and published examples of transparent reporting. The STROBE checklist is best used in conjunction with this article (freely available on the Web sites of PLoS Medicine at http://www.plosmedicine.org/, Annals of Internal Medicine at http://www.annals.org/, and Epidemiology at http://www.epidem.com/). Information on the STROBE Initiative is available at www.strobe-statement.org.

For peer review only

For peer review only - http://bmjopen.bmj.com/site/about/guidelines.xhtml

BMJ Open

Self-reported sleep duration and napping, cardiac risk factors and markers of subclinical vascular disease: cross-sectional study in older men

Journal:	*BMJ Open*
Manuscript ID	bmjopen-2017-016396.R2
Article Type:	Research
Date Submitted by the Author:	10-May-2017
Complete List of Authors:	Zonoozi, Shahrzad; UCL, Department of Primary Care & Population Health<br>Ramsay, Sheena; Newcastle University, Institute of Health & Society<br>Papacosta, Olia; University College London,<br>Lennon, Lucy; UCL, Department of Primary Care and Population Health<br>Ellins, Elizabeth; Swansea University, Institute of Life Sciences<br>Halcox, Julian; Swansea University, Department of Cardiology<br>Whincup, Peter; St George's University of London,<br>Wannamethee, Goya; UCL, Primary Care and Population Health
**Primary Subject Heading**:	Epidemiology
Secondary Subject Heading:	Cardiovascular medicine
Keywords:	Sleep patterns, Cardiac function, Subclinical atherosclerosis

SCHOLARONE™
Manuscripts

For peer review only

Self-reported sleep duration and napping, cardiac risk factors and markers of subclinical vascular disease: cross-sectional study in older men

Shahrzad Zonoozi[1]; Sheena E Ramsay[2]; Olia Papacosta[1]; Lucy Lennon[1]; Elizabeth A Ellins[3]; Julian P J Halcox[3]; Peter H Whincup[4]; S Goya Wannamethee[1]

[1] UCL Department of Primary Care & Population Health, UCL Medical School, Rowland Hill Street, London, NW3 2PF, UK

[2] Institute of Health & Society, Newcastle University, Richardson Road, Newcastle upon Tyne, NE2 4AX, UK

[3] Institute of Life Sciences, Swansea University, Singleton Park, Swansea, SA2 8PP, UK

[4] Population Health Research Institute, St George's University of London, Cranmer Terrace, London, SW17 0RE, UK

Corresponding author:

Shahrzad Zonoozi, UCL Department of Primary Care & Population Health, UCL Medical School, Rowland Hill Street, London, NW3 2PF, UK. Email: shahrzadz@gmail.com

Word count: 3595; 4 tables

Keywords: sleep patterns, cardiac function, subclinical atherosclerosis

For peer review only - http://bmjopen.bmj.com/site/about/guidelines.xhtml

For peer review only

Abstract

Study Objectives: Daytime sleep has been associated with increased risk of cardiovascular disease and heart failure (HF) but the mechanisms remain unclear. We have investigated the association between daytime and night-time sleep patterns and cardiovascular risk markers in older adults including cardiac markers and subclinical markers of atherosclerosis [arterial stiffness and carotid intima-media thickness (CIMT)].

Methods: Cross sectional study of 1722 surviving men aged 71-92 examined in 2010-2012 across 24 British towns, from a prospective study initiated in 1978-1980. Participants completed a questionnaire and were invited for a physical examination. Men with a history of heart attack or HF (n = 251) were excluded from the analysis.

Results: Self-reported daytime sleep duration was associated with higher fasting glucose and insulin levels (p = 0.02 and 0.01 respectively) even after adjustment for age, BMI, physical activity and social class. Compared to those with no daytime sleep, men with daytime sleep >1 hour, defined as excessive daytime sleepiness (EDS), had a higher risk of raised N-terminal pro brain natriuretic peptide (NT-pro BNP) of ≥400pg/ml, the diagnostic threshold for HF [OR (95% CI) = 1.88 (1.15, 3.1)], higher mean troponin (cTnT), reduced lung function (forced expiratory volume in 1s) and elevated von Willebrand factor (vWF), a marker of endothelial dysfunction. However, EDS was unrelated to CIMT and arterial stiffness. By contrast night-time sleep was only associated with HbA1c (short or long sleep) and arterial stiffness (short sleep).

Conclusions: Daytime sleep duration of >1 hour may be an early indicator of HF.

Word count: 248

For peer review only - http://bmjopen.bmj.com/site/about/guidelines.xhtml

Article Summary

Strength and limitations of this study

- To the best of our knowledge, this is the first study to look at he association between excessive daytime sleepiness (EDS), cardiac markers and vascular parameters.
- Owing to a lack of data, we do not know if the associations seen are true for women, middle aged populations and ethnic minorities.
- As we do not have polysomnography data, we were unable to fully exclude obstructive sleep apnoea as a contributing factor to our findings.
- Our results are based on a cross-sectional analysis and therefore causality cannot be established.

For peer review only

For peer review only - http://bmjopen.bmj.com/site/about/guidelines.xhtml

Introduction

Sleep disturbance is a common complaint, especially amongst older adults. (1) It is increasingly recognised that there is a U shaped association between sleep duration and risk of cardiovascular disease (CVD) and metabolic disturbances – i.e. both short and long sleep durations are associated with an increased risk. (2-4) More recently, studies have shown excessive daytime sleepiness (EDS), one of the more common sleep disturbances reported in the older population, to be associated with increased risks of CVD (5, 6), heart failure (HF) (6, 7) and all-cause mortality. (8-10)

The mechanism of the association between EDS and CVD risk remains unclear. Whilst some studies have looked at the association between EDS and metabolic risk markers (11, 12), few have examined mechanisms by which EDS may influence CVD and in particular incident HF. (6) In particular, the association between EDS and markers of arterial disease and surrogate markers of cardiac function, including NT-pro brain natriuretic peptide (BNP), a marker of ventricular stress, and cardiac troponin T (cTnT), a marker of myocardial injury, have been less studied. Sub-clinical arterial disease markers are increasingly being used as early predictors for atherosclerosis and CVD. A number of non-invasive assessments have been developed, including measures of atherosclerosis such as carotid intima-media thickness (CIMT) and indirect measures of arterial stiffness including carotid distensibility and pulse wave velocity (PWV). Although a number of studies have examined the association between night-time sleep duration and arterial stiffness, (13-17) studies on the association between EDS and vascular risk markers are limited. (5) We have therefore examined the association between daytime sleep as well as night-time sleep with vascular risk factors including metabolic risk factors, inflammatory and endothelial markers, cardiac markers and measures of sub-clinical arterial disease (including arterial stiffness and CIMT).

For peer review only - http://bmjopen.bmj.com/site/about/guidelines.xhtml

Methods

The British Regional Heart Study is a prospective study which recruited a socioeconomically and geographically representative cohort of 7735 men from 24 British towns between 1978 and 1980. The present investigation is based on a reassessment in 2010-2012, when all surviving men (n = 3137), aged between 71–92 years, were sent a postal questionnaire and invited for a 30th year re-examination. 2137 (68%) men completed the postal questionnaire and 1722 (55%) men attended the re-examination. (18) Blood samples were collected after fasting for a minimum of six hours and were stored at −70°C. Ethical approval for data collection was obtained from the relevant research ethics committee. The men were asked if a doctor had ever told them they had a heart attack or HF and these men were excluded from the analysis (n = 251). Of the men with no history of heart attack or HF, 1471 men had physical, blood and ultrasound measurements carried out. This present study is a cross-sectional analysis of the data from the 2010-2012 questionnaires and re-examination.

CVD risk factors: Physical examination included blood pressure (BP), lung function as well as height and weight, from which body mass index (BMI) was calculated. (19) Height standardised forced expiratory volume in 1 second (FEV1) was measured using the Vitalograph Compact II. Details of measurement and classification for smoking status and physical activity in this cohort have been previously described. (20) Details of the men's medication history, including use of antihypertensive, hypnotic and anxiolytic medication, were recorded at the examination and participants were also asked to report symptoms of breathlessness. Measurements of metabolic, inflammatory and endothelial markers were taken as described previously. (19, 21, 22) NT-pro BNP and cTnT were measured using the Elecsys 2010 electrochemiluminescence method (Roche Diagnostics, Burgess Hill, UK). The lowest detectable value of cTnT was 5 and for anyone with a recorded reading of < 5, we used half of the lowest detectable value (i.e. 2.5). NT-pro BNP ≥400pg/ml was defined as high, as per current guidelines issued by the National Institute of Clinical Excellence (NICE)

For peer review only - http://bmjopen.bmj.com/site/about/guidelines.xhtml

for the diagnosis of HF. (23) Prevalent diabetes included men with doctor-diagnosed diabetes and men with fasting blood glucose ≥7 mmol/L. 1402 men had at least one blood measurement (69 missing). Chronic kidney disease (CKD) was defined as estimated glomerular filtration rate of less than 60 using the CKD Epidemiology Collaboration (CKD-EPI) creatinine equation. (24)

Sleep parameters: Reported duration of night sleep was based on response to: "On average, how many hours of sleep do you have each night?" Duration of day sleep was based on response to "On average, how much sleep (if any) do you have during the daytime?" If a response was entered for the night sleep question but no response was indicated for the day sleep question, the answer was set to 0. 1436 men completed the questions on night-time and daytime sleep duration (35 missing). Night-time sleep was categorised into three groups: <6, 6-8.9 and ≥9 hours. Daytime sleep was categorised into four groups: 0 hours, <1 hour, 1 hour and >1 hour. Those sleeping >1 hour during the daytime were defined as having EDS.

Non-invasive markers of arterial disease: Measurements were measured by two vascular technicians in series. Left and right carotid arteries were images using a Z.One Ultra ultrasound system (Zonare Medical Systems, Mountain View, CA) with a 5-10-mHz linear probe. A cross sectional sweep from the base of the common carotid artery to the jaw bone and longitudinal images of the common carotid artery approximately 1cm proximal to the carotid bifurcation were recorded. Peak systolic and end-diastolic common carotid artery diameter and CIMT, the distance between the leading edge of the intima and the media-adventitia interface, were measured using Carotid Analyser software (Medical Imaging Applications, Iowa City, IA). From the longitudinal images, a region of interest (5-10 mm) was selected in a plaque free area, at least 1cm from the bifurcation.

CIMT was measured from three end-diastolic images on each side and a mean of these measures was calculated. Maximum and minimum carotid artery diameter was assessed from three consecutive waveforms and mean distension was calculated as: maximum

For peer review only - http://bmjopen.bmj.com/site/about/guidelines.xhtml

diameter – minimum diameter. The distensibility coefficient was then calculated as described by Dijk *et al.*: distensibility coefficient = [(2 x mean distension/baseline diameter)/mean pulse pressure (kPa)]*1000. (25) Carotid to femoral PWV was assessed using a Vicorder (Skidmore Medical, Bristol UK), with participants in a semi-supine position with their torso at approximately 30˚. A 2x9cm cuff was positioned around the neck with the bladder over the right carotid pulse, and a Hokanson SC10 cuff around the middle of the right thigh. Path length was measured from the sternal notch to the centre of the thigh cuff. The cuffs were simultaneously inflated and traces with a minimum of 3 good quality waveforms recorded. Two PWV measurements, within ≤0.5m/s of each other, were accepted and averaged. 1464 men had at least one non-invasive vascular measurement carried out (7 missing).

Statistical analysis: Distributions of HbA1c, glucose, insulin, CRP, IL-6, NT-pro BNP and cTnT were highly skewed and log transformation was used. Comparisons of baseline characteristics between the sleep groups were carried out using the chi squared test for categorical variables and analysis of variance for continuous variables. Logistic regression models were used to assess the differences in outcomes between categorical groups. In multivariate analyses, age, BMI, physical activity and social class were fitted as continuous variables whilst prevalent diabetes and use of antihypertensive medications was defined as categorical variables. The base groups for the statistical analysis were the group sleeping 6-8.9 hours in the night-time sleep group. A threshold effect was seen in those sleeping >1 hour in the daytime and therefore we compared those sleeping >1 hour with those sleeping ≤1. We also carried out statistical analysis of the trend in the daytime sleep group. All analyses were performed using SAS version 9.3 (SAS, Cary, North Carolina).

Results

The average duration of night-time sleep in the men who responded was 6.8 hours (± 1.4 hours). 15% of the men reported night-time sleep duration of less than 6 hours, while 7% reported sleeping ≥9 hours during the night. 47% of men reported no daytime sleep whilst

For peer review only - http://bmjopen.bmj.com/site/about/guidelines.xhtml

9% reported EDS. Night-time sleep duration was significantly associated with EDS (p = 0.02; correlation coefficient r = -0.063), with a smaller proportion of those sleeping ≥9 hours reporting EDS. Daytime sleep duration was significantly associated with night-time sleep of less than 6 hours (p = 0.02).

Baseline characteristics

Table 1 describes the baseline characteristics of the study population according to night-time and daytime sleep patterns. A U-shaped relationship was seen between night-time sleep and CVD risk factors. Both short and long night-time sleep were associated with increased age, increased prevalence of physical inactivity, manual workers and breathlessness compared to those with 6-8.9 hours sleep. There was a significant association between night-time sleep and use of hypnotic or anxiolytic medications. Daytime sleep is strongly associated with older age and with many adverse characteristics including physical inactivity, obesity, prevalent diabetes, breathlessness, use of anti-hypertensive medication, depression and CKD. The prevalence of these adverse factors tended to increase with increasing duration of daytime sleep. Daytime sleep was not associated with use of hypnotic or anxiolytic medications.

Night-time sleep and and cardiovascular risk markers

There was a U-shaped association between night-time sleep and metabolic risk factors including HbA1c and glucose (Table 2). The association between night-time sleep and HbA1c remained significant following adjustment for daytime sleep (p = 0.04). In age-adjusted analysis, night-time sleep was significantly associated with IL-6, a marker of inflammation, but this association was attenuated following adjustment for BMI, social class and physical activity (p = 0.05). No significant association was seen between night-time

For peer review only - http://bmjopen.bmj.com/site/about/guidelines.xhtml

sleep and von Willebrand factor (vWF), a marker of endothelial function, FEV1 (lung function) or cardiac markers (NT-proBNP and cTnT).

Daytime sleep and cardiovascular risk markers

In age-adjusted analysis, metabolic risk factors including HDL-C, triglycerides, HbA1c and insulin tended to increase with increasing hours of daytime sleep (Table 3). No association was seen with BP. The increasing trend was attenuated after adjustment for age, BMI, social class and physical activity. However compared to those who slept $\leq$1 hour, those with EDS (>1 hour) showed significantly higher mean levels of HbA1c, glucose and insulin (Table 3). Mean levels of glucose and insulin remained significantly higher in those with EDS (p = 0.03 and 0.01 respectively) even after further adjustment for prevalent diabetes and night-time sleep.

CRP increased with increasing daytime sleep duration, even after full adjustment (p trend = 0.04). Lung function decreased with increasing daytime sleep duration with levels markedly reduced in those with EDS compared to those reporting $\leq$1 hour daytime sleep (p < 0.0001). Only EDS was associated with significantly higher mean vWF levels (p = 0.03). When we examined the odds of having high vWF (top quintile) and low FEV1 (lowest quintile), those with EDS were 1.8 times more likely to have a high vWF [OR (95% CI) = 1.82 (1.17, 2.83)] and 2.4 times more likely to have a low FEV1 [OR (95% CI) = 2.4 (1.53, 3.74)] compared to those who reported no daytime sleep.

Mean levels of NT-pro BNP and cTnT (cardiac markers) were significantly higher only in those with EDS following full adjustment (p = 0.02 and 0.03 respectively). This association remained significant even after adjustment for CKD. Men with EDS were more likely to have high (top quintile) mean NT-pro BNP [OR (95% CI) = 1.69 (1.06, 2.7)] and cTnT [OR (95% CI) = 1.75 (1.09, 2.8)] compared to those who reported no daytime sleep. However, this was attenuated following adjustment for prevalent diabetes, use of antihypertensive medication and depression [OR (95% CI) = 1.59 (0.99, 2.55)] and [OR (95% CI) = 1.62 (1.00, 2.62)]

For peer review only - http://bmjopen.bmj.com/site/about/guidelines.xhtml

respectively, although it remained significant for cTnT. We further looked at men with a NT-pro BNP of ≥400pg/ml, the diagnostic threshold for HF. Men who reported EDS had a higher odds of having an NT-pro BNP of ≥400pg/ml [OR (95% CI) = 1.88 (1.15, 3.1)]. This association remained following adjustment for prevalent diabetes, CKD, use of antihypertensive medication and depression [OR (95% CI) = 1.72 (1.03, 2.87)]. Exclusion of men using anxiolytic and hypnotic medications did not significantly alter this [OR (95% CI) = 1.76 (1.05, 2.95)].

Sleep patterns and non-invasive markers of arterial disease

Table 4 shows the associations between sleeping patterns and non-invasive vascular measurements. There was a significant association between those sleeping less than 6 hours and mean PWV and distensibility when compared to those sleeping 6-8.9 hours in the fully adjusted model (p = 0.04 and 0.03 respectively). Men who slept less than 6 hours during the night-time were 1.6 times more likely to have a low (lowest quintile) mean distensibility compared to those who slept 6-8.9 hours in the fully adjusted model [OR (95% CI) = 1.56 (1.09, 2.23)]. There was no significant association between daytime sleep and markers of arterial stiffness. There was no association between sleeping patterns and CIMT in the study population.

For peer review only - http://bmjopen.bmj.com/site/about/guidelines.xhtml

Discussion

We have confirmed previous reports of an association between night-time sleep duration (4) and daytime sleep duration (11, 12) with metabolic risk factors. Our findings extend previous studies by investigating the association between EDS and a wide range of cardiac markers and vascular measures not previously examined. Our study has shown that in a representative sample of British men aged 71–92 years with no prior history of heart attack or HF, those who sleep more than one hour during the day have a number of adverse characteristics including higher mean levels of cardiac markers, metabolic risk factors, inflammatory and endothelial markers and reduced lung function. These findings may point to pathways for the association observed between EDS and CVD and all-cause mortality. (8)

EDS and metabolic and cardiac risk markers

EDS, defined in our study as daytime sleep of >1 hour, is associated with many adverse characteristics such as obesity, physical inactivity, diabetes, breathlessness, use of antihypertensive therapy and with an adverse pattern of metabolic risk factors including HbA1c, glucose and insulin. Our results confirm previous cross-sectional analyses which have shown EDS to be associated with the metabolic syndrome and longer habitual napping duration to be associated with an increased risk of impaired fasting glucose and diabetes mellitus. (11, 12) There was a significant linear relationship between increasing daytime sleep and mean CRP levels, with those reporting EDS having higher levels of mean CRP. This confirms results from a previous study which showed self-reported daytime napping is associated with higher levels of CRP in older British adults. (26) The association seen between daytime sleep and these markers of inflammation may provide an explanation for the excess all-cause mortality observed in those with daytime napping or EDS. (8, 9) Whilst both of these studies have adjusted for diabetes, neither has adjusted for inflammation.

We found a significant association between EDS and high levels of NT-pro BNP and cTnT, both of which are biomarkers of HF. (27, 28) NT-pro BNP is produced in response to cardiac

For peer review only - http://bmjopen.bmj.com/site/about/guidelines.xhtml

wall stress and is a strong predictor of HF. (22) Troponin T is a plasma marker of myocyte necrosis, which has been positively associated with incident CVD, HF and all-cause mortality. (29) Most notably men who reported EDS had a higher odds of having NT-pro BNP of ≥400pg/ml (the diagnostic threshold for HF). As far as we are aware, this is the first study to look at the association between EDS and these cardiac markers.

A strong association was also seen between EDS and elevated vWF (marker of endothelial dysfunction) and low FEV1, markers associated with HF. These findings suggest that EDS may be an early indicator of HF. This supports a recent report from our study when the men were aged 63-82 years which showed EDS to be associated with the risk of developing overt HF during 9 year follow-up. (7) In that analyses cardiac markers and lung function were not available.

The association between EDS and HF may be explained by underlying respiratory illness such as obstructive sleep apnoea (OSA). Indeed within our cohort, EDS was significantly associated with obesity, self-reported breathlessness and reduced lung function. EDS may be a result of disturbed night-time sleep and difficulty falling asleep, both of which have been associated with HF. (30, 31) However, the associations we observed with cardiac markers and lung function were not seen in men with a short or long duration of night-time sleep and further analyses showed no association between these markers and those reporting difficulty falling asleep.

Non-invasive markers of arterial disease

In contrast to night-time sleep we did not observe any significant association between daytime sleep and markers of arterial stiffness. Short night-time sleep but not long night-time sleep was associated with arterial stiffness (PWV and carotid distensibility), when compared to those sleeping 6-8.9 hours. There was no association between sleeping patterns and CIMT in the study population. This is in contrast to previous studies which have shown an

For peer review only - http://bmjopen.bmj.com/site/about/guidelines.xhtml

For peer review only

association between short night-time sleep (17) and EDS (5) with CIMT. The differences observed may be due to use of brachial-ankle PWV (13, 15), rather than carotid-femoral PWV, different classification of long night-time sleep (13, 14) and a younger study population. (15)

Strengths and limitations

The strengths of our study include the fact that it is a study of older men, a group who are at high risk of vascular disease and the wide range of vascular parameters which we have looked into. Limitations of our study include the lack of representation of women, middle aged populations and ethnic minority groups which limits generalisability. Our data is based on self-reported sleeping patterns at a single time point and it is possible that night-time and daytime sleep duration have been misclassified. Our analysis of night-time sleep relies on average duration of night-time sleep and we do not have measures of interruption or total time spent in bed. As we do not have polysomnography data, we were unable to fully exclude OSA as a contributing factor to our findings. Data regarding occupation was not available and we were unable to assess if previous shift work, which has been reported to be associated with cardiovascular and metabolic diseases, is related to the adverse cardiovascular profile we noted in those with EDS. We appreciate that our results are based on a cross-sectional analysis and therefore causality cannot be established and the direction of the effect is unknown. It is also possible that there is an element of survival bias in our data; it may be that men who attended the re-examination were healthier and this may account for the null associations seen.

Conclusions

EDS, defined as daytime sleep of more than one hour, is associated with an adverse cardiovascular profile and may be an early indicator of HF. Clinically, this is important in the

For peer review only - http://bmjopen.bmj.com/site/about/guidelines.xhtml

assessment of older patients who seek medical advice for EDS, in whom indicators of HF should be sought and further investigations carried out if necessary.

Disclosures

The authors report no conflicts of interest.

Funding

The British Regional Heart Study is a British Heart Foundation (BHF) research group. This work was supported by a British Heart Foundation programme grant (RG/13/16/30528) and project grant (PG/09/024, PG/13/41/30304).

Contributions

SGW initiated the concept and design of the paper. SZ analysed the data and drafted the manuscript. SGW, JPJH, EAE, PHW contributed to the interpretation of data. OP contributed to the analysis of the paper. EAE, JPJH, LL, PHW and SGW contributed to the acquisition of the data. All authors revised it critically for important intellectual content and approved the final version of the manuscript.

Data Sharing Statement

Further details of the Study can be found on http://www.ucl.ac.uk/pcph/research-groups-themes/brhs-pub.

For general data sharing enquiries, please contact Lucy Lennon (l.lennon@ucl.ac.uk).

For peer review only

For peer review only - http://bmjopen.bmj.com/site/about/guidelines.xhtml

Table 1 - Baseline characteristics of the study population

		Age mean ±std	Obese (%)	Cigarette Smoker (%)	Inactive (%)	Manual social class (%)	Diabetic (%)	Breathlessness (%)	Using Antihypertensives (%)	Depression (%)	CKD (%)
**Night-time Sleep Duration (hours)**	**< 6 (n = 213[a])**	78.76±4.6	21.53	0.94	48.36	57.35	18.31	40.72	53.99	11.43	29
	**6-8.9 (n = 1126[a])**	78.22±4.6	18.78	3.83	33.66	42.4	14.87	19.32	48.85	8.39	29.36
	**≥ 9 (n = 97[a])**	79.24±4.9	22.11	4.12	38.14	48.45	19.79	25	52.58	11.34	38.71
	**p**	0.05	0.52	0.10	0.0002 *	0.002 *	0.24	<.0001 *	0.33	0.27	0.16
**Daytime Sleep Duration (hours)**	**0 (n = 680[a])**	77.83±4.4	16.1	2.65	30.74	46.1	12.24	17.59	45.88	7.41	26.42
	**<1 (n = 290[a])**	78.04±4.4	15.63	2.76	33.45	40	14.88	21.61	54.14	10.42	30.94
	**1 (n = 336[a])**	78.79±4.7	24.17	5.07	40.77	44.61	20	27.01	49.7	8.98	32.81
	**>1 (n = 130[a])**	80.05±5	33.33	4.62	58.46	51.54	24.62	41.46	61.54	14.62	39.17
	**p**	<.0001 *	<.0001 *	0.18	<.0001 *	0.15	0.0003 *	<.0001 *	0.004 *	0.05	0.02 *

a, maximum n in group, varies slightly with missing co-variate data

CKD, chronic kidney disease

For peer review only - http://bmjopen.bmj.com/site/about/guidelines.xhtml

For peer review only

Table 2 - Night-time sleep duration vs metabolic risk factors, inflammatory and endothelial markers, lung function and cardiac markers.

		Night Sleep (hours)			
		< 6 (n = 213[d])	6-8.9 (n = 1126[d])	≥ 9 (n = 97[d])	p
**Metabolic risk factors [mean (95% CI)]**					
**SBP (mmHg)**	**A**	146.04 (143.52, 148.56)	147.31 (146.21, 148.4)	150.73 (146.99, 154.47)	0.12
**DBP (mmHg)**	**A**	76.59 (75.07, 78.11)	77.46 (76.8, 78.12)	77.25 (74.99, 79.5)	0.59
**HDL-C (mmol/L)**	**A**	1.47 (1.41, 1.53)	1.48 (1.45, 1.5)	1.37 (1.28, 1.46)	0.07
**Triglycerides (mmol/L)**	**A**	1.3 (1.21, 1.39)	1.28 (1.25, 1.32)	1.39 (1.26, 1.52)	0.31
**HbA1c[c] (%)**	**A**	5.93 (5.85, 6.02)	5.75 (5.72, 5.79)	5.88 (5.76, 6)	0.0001 *
	**B**	5.9 (5.82, 5.98)	5.76 (5.73, 5.79)	5.86 (5.74, 5.97)	0.003 *
**Glucose[c] (mmol/L)**	**A**	5.78 (5.62, 5.94)	5.55 (5.48, 5.61)	5.72 (5.49, 5.95)	0.02 *
	**B**	5.74 (5.58, 5.9)	5.56 (5.49, 5.62)	5.68 (5.45, 5.91)	0.08
**Insulin[c] (mU/L)**	**A**	8.26 (7.54, 9.04)	7.92 (7.62, 8.24)	7.82 (6.84, 8.94)	0.69
**Inflammatory and endothelial markers [mean (95% CI)]**					
**CRP[c] (mg/L)**	**A**	1.51 (1.28, 1.78)	1.34 (1.25, 1.44)	1.46 (1.15, 1.87)	0.37
**IL-6[c] (pg/mL)**	**A**	3.03 (2.74, 3.35)	2.98 (2.86, 3.11)	3.72 (3.21, 4.3)	0.02 *
	**B**	2.94 (2.67, 3.25)	3 (2.87, 3.13)	3.61 (3.12, 4.18)	0.05
**vWF (IU/dL)**	**A**	140.97 (130.97, 150.97)	133.27 (128.91, 137.63)	119.04 (104.2, 133.89)	0.06
**Lung function [mean (95% CI)]**					
**FEV1 (L)**	**A**	2.43 (2.35, 2.5)	2.46 (2.42, 2.49)	2.44 (2.32, 2.55)	0.76
**Cardiac markers [mean (95% CI)]**					
**NT-pro BNP[c] (pg/mL)**	**A**	121.67 (101.33, 146.1)	126.45 (116.87, 136.81)	134.61 (102.8, 176.25)	0.83
**cTnT[c] (pg/ml)**	**A**	11.25 (10.29, 12.3)	10.82 (10.41, 11.24)	10.14 (8.89, 11.57)	0.44

A, adjusted for age

B, adjusted for age, BMI, social class and physical activity

c, geometeric mean

d, maximum n in group, varies slightly with missing co-variate data

SBP, systolic blood pressure; DBP, diastolic blood pressure; HDL-C, high density lipoprotein cholesterol; HbA1c, glycated haemoglobin; CRP, C-reactive protein; IL-6, interleukin 6; vWF, von Willebrand factor; FEV1, forced expiratory volume in 1s; NT-pro BNP, N-terminal pro brain natriuretic peptide; cTnT, cardiac troponin T

For peer review only

For peer review only - http://bmjopen.bmj.com/site/about/guidelines.xhtml

Table 3 – Daytime sleep duration vs metabolic risk factors, inflammatory and endothelial markers, lung function and cardiac markers.

		Daytime Sleep Duration (Hours)					
		0 (n = 680[d])	<1 (n = 290[d])	1 (n = 336[d])	>1 (n = 130[d])	p value trend	p value >1h vs others
**Metabolic risk factors [mean (95% CI)]**							
SBP (mmHg)	A	147.46 (146.05, 148.88)	148.76 (146.59, 150.92)	147.14 (145.13, 149.15)	144.13 (140.88, 147.39)	0.90	0.04 *
	B	147.49 (146.07, 148.9)	148.59 (146.42, 150.75)	146.97 (144.95, 148.99)	144.78 (141.44, 148.11)	0.96	0.12
DBP (mmHg)	A	77.41 (76.56, 78.26)	78.08 (76.77, 79.38)	77.16 (75.95, 78.37)	75.54 (73.57, 77.5)	0.98	0.06
	B	77.56 (76.7, 78.41)	78.04 (76.74, 79.35)	76.94 (75.73, 78.16)	75.64 (73.63, 77.65)	0.74	0.09
HDL-C (mmol/L)	A	1.5 (1.47, 1.54)	1.47 (1.42, 1.52)	1.44 (1.39, 1.49)	1.33 (1.25, 1.41)	0.01 *	0.0002 *
	B	1.49 (1.46, 1.52)	1.46 (1.41, 1.5)	1.46 (1.42, 1.51)	1.41 (1.33, 1.48)	0.14	0.10
Triglycerides (mmol/L)	A	1.24 (1.19, 1.29)	1.3 (1.23, 1.38)	1.35 (1.28, 1.42)	1.42 (1.31, 1.54)	0.01 *	0.02 *
	B	1.26 (1.21, 1.3)	1.33 (1.26, 1.4)	1.33 (1.26, 1.4)	1.34 (1.22, 1.45)	0.03 *	0.47
HbA1c[c] (%)	A	5.73 (5.69, 5.78)	5.74 (5.68, 5.81)	5.87 (5.8, 5.93)	5.99 (5.88, 6.1)	0.01 *	0.0003 *
	B	5.75 (5.7, 5.79)	5.77 (5.7, 5.83)	5.84 (5.78, 5.91)	5.9 (5.79, 6.01)	0.04 *	0.04 *
Glucose[c] (mmol/L)	A	5.52 (5.43, 5.6)	5.48 (5.36, 5.61)	5.7 (5.58, 5.83)	5.98 (5.77, 6.2)	0.09	0.0002 *
	B	5.54 (5.45, 5.62)	5.51 (5.38, 5.63)	5.68 (5.56, 5.8)	5.86 (5.65, 6.07)	0.25	0.01 *
Insulin[c] (mU/L)	A	7.5 (7.13, 7.88)	7.43 (6.89, 8.02)	8.69 (8.09, 9.33)	10.36 (9.21, 11.65)	0.01 *	<.0001 *
	B	7.7 (7.36, 8.07)	7.68 (7.16, 8.24)	8.31 (7.77, 8.88)	9.29 (8.32, 10.37)	0.15	0.005 *
**Inflammatory and endothelial markers [mean (95% CI)]**							
CRP[c] (mg/L)	A	1.24 (1.13, 1.36)	1.36 (1.19, 1.57)	1.56 (1.37, 1.78)	1.67 (1.35, 2.08)	0.01 *	0.07
	B	1.27 (1.16, 1.39)	1.4 (1.22, 1.61)	1.5 (1.32, 1.71)	1.51 (1.21, 1.89)	0.04 *	0.37
IL-6[c] (pg/mL)	A	2.88 (2.73, 3.05)	3.01 (2.76, 3.28)	3.19 (2.95, 3.45)	3.58 (3.14, 4.09)	0.03 *	0.01 *
	B	2.92 (2.76, 3.08)	3.06 (2.81, 3.33)	3.13 (2.89, 3.39)	3.34 (2.92, 3.81)	0.10	0.15
vWF (IU/dL)	A	132.65 (127.06, 138.24)	127.34 (118.81, 135.87)	134.92 (126.85, 142.98)	148.03 (135.05, 161.01)	1.00	0.02 *
	B	132.76 (127.1, 138.41)	127.64 (119.03, 136.24)	134.88 (126.7, 143.07)	147.74 (134.39, 161.1)	0.96	0.03 *
**Lung function [mean (95% CI)]**							
FEV1 (L)	A	2.52 (2.48, 2.57)	2.46 (2.39, 2.52)	2.37 (2.31, 2.44)	2.21 (2.11, 2.31)	<.0001 *	<.0001 *
	B	2.52 (2.48, 2.56)	2.44 (2.38, 2.51)	2.38 (2.32, 2.44)	2.26 (2.15, 2.36)	<.0001 *	<.0001 *
**Cardiac markers [mean (95% CI)]**							
NT-pro BNP[c] (pg/mL)	A	122.31 (110.53, 135.34)	110.95 (95.15, 129.37)	135.15 (117.08, 156.02)	171.15 (134.5, 217.78)	0.70	0.01 *
	B	123.28 (111.4, 136.43)	111.2 (95.39, 129.62)	135.76 (117.52, 156.82)	167.33 (130.92, 213.88)	0.82	0.02 *
cTnT[c] (pg/ml)	A	10.33 (9.83, 10.85)	10.45 (9.7, 11.27)	11.59 (10.8, 12.43)	12.79 (11.37, 14.38)	0.03 *	0.004 *
	B	10.41 (9.91, 10.93)	10.52 (9.77, 11.33)	11.31 (10.55, 12.13)	12.3 (10.93, 13.86)	0.12	0.03 *

For peer review only - http://bmjopen.bmj.com/site/about/guidelines.xhtml

A, adjusted for age

B, adjusted for age, BMI, social class and physical activity

c, geometeric mean

d, maximum n in group, varies slightly with missing co-variate data

SBP, systolic blood pressure; DBP, diastolic blood pressure; HDL-C, high density lipoprotein cholesterol; HbA1c, glycated haemoglobin; CRP, C-reactive protein; IL-6, interleukin 6; vWF, von Willebrand factor; FEV1, forced expiratory volume in 1s; NT-pro BNP, N-terminal pro brain natriuretic peptide; cTnT, cardiac troponin T

For peer review only

For peer review only - http://bmjopen.bmj.com/site/about/guidelines.xhtml

Table 4 – Sleeping patterns vs non-invasive vascular measurements.

		CIMT (mm)		PWV (m/s)		Distensibility ($10^{-3}kPa^{-1}$)	
		**Age-adjusted**	**Additional Adj[a]**	**Age-adjusted**	**Additional Adj[a]**	**Age-adjusted**	**Additional Adj[a]**
		**mean (95% CI)**	**mean (95% CI)**	**mean (95% CI)**	**mean (95% CI)**	**mean (95% CI)**	**mean (95% CI)**
**Night-time Sleep Duration (hours)**	**< 6 (n = 209[b])**	0.81 (0.78, 0.83)	0.8 (0.78, 0.82)	10.48 (10.24, 10.71)	10.45 (10.22, 10.69)	11.66 (11.11, 12.21)	11.62 (11.06, 12.18)
	**6-8.9 (n = 1125[b])**	0.81 (0.8, 0.82)	0.81 (0.8, 0.82)	10.18 (10.08, 10.27)	10.18 (10.08, 10.28)	12.29 (12.06, 12.53)	12.31 (12.08, 12.55)
	**≥ 9 (n = 95[b])**	0.82 (0.79, 0.85)	0.82 (0.79, 0.85)	10.25 (9.91, 10.6)	10.25 (9.9, 10.59)	11.71 (10.9, 12.51)	11.81 (11, 12.63)
	**p**	0.67	0.64	0.07	0.11	0.06	0.06
**Daytime Sleep Duration (hours)**	**0 (n = 677[b])**	0.81 (0.79, 0.82)	0.81 (0.8, 0.82)	10.25 (10.12, 10.38)	10.25 (10.12, 10.38)	12.23 (11.93, 12.53)	12.2 (11.89, 12.5)
	**<1 (n = 290[b])**	0.81 (0.79, 0.82)	0.81 (0.79, 0.82)	10.15 (9.96, 10.35)	10.16 (9.96, 10.36)	12.49 (12.03, 12.96)	12.5 (12.04, 12.97)
	**1 (n = 334[b])**	0.8 (0.79, 0.82)	0.8 (0.78, 0.82)	10.2 (10.01, 10.38)	10.2 (10.01, 10.38)	11.81 (11.38, 12.25)	11.88 (11.44, 12.32)
	**>1 (n = 128[b])**	0.84 (0.81, 0.87)	0.83 (0.8, 0.86)	10.29 (9.99, 10.6)	10.29 (9.98, 10.6)	11.96 (11.25, 12.67)	12.12 (11.39, 12.85)
	**p**	0.17	0.37	0.80	0.85	0.18	0.30

a, adjusted for age, BMI, social class and physical activity

b, maximum n in group

CIMT, carotid intima-media thickness; PWV, pulse wave velocity

For peer review only

Bibliography

1. Flamer HE. Sleep disorders in the elderly. Australian and New Zealand Journal of Medicine. 1996;26(1):96-104.
2. Grandner MA, Sands-Lincoln MR, Pak VM, Garland SN. Sleep duration, cardiovascular disease, and proinflammatory biomarkers. Nature and science of sleep. 2013;5:93-107.
3. Jackson CL, Redline S, Emmons KM. Sleep as a potential fundamental contributor to disparities in cardiovascular health. Annual review of public health. 2015;36:417-40.
4. Knutson KL. Sleep duration and cardiometabolic risk: a review of the epidemiologic evidence. Best practice & research Clinical endocrinology & metabolism. 2010;24(5):731-43.
5. Blachier M, Dauvilliers Y, Jaussent I, Helmer C, Ritchie K, Jouven X, et al. Excessive daytime sleepiness and vascular events: the Three City Study. Annals of neurology. 2012;71(5):661-7.
6. Newman AB, Spiekerman CF, Enright P, Lefkowitz D, Manolio T, Reynolds CF, et al. Daytime sleepiness predicts mortality and cardiovascular disease in older adults. The Cardiovascular Health Study Research Group. Journal of the American Geriatrics Society. 2000;48(2):115-23.
7. Wannamethee SG, Papacosta O, Lennon L, Whincup PH. Self-Reported Sleep Duration, Napping, and Incident Heart Failure: Prospective Associations in the British Regional Heart Study. Journal of the American Geriatrics Society. 2016.
8. Leng Y, Wainwright NW, Cappuccio FP, Surtees PG, Hayat S, Luben R, et al. Daytime napping and the risk of all-cause and cause-specific mortality: a 13-year follow-up of a British population. American journal of epidemiology. 2014;179(9):1115-24.
9. Tanabe N, Iso H, Seki N, Suzuki H, Yatsuya H, Toyoshima H, et al. Daytime napping and mortality, with a special reference to cardiovascular disease: the JACC study. International journal of epidemiology. 2010;39(1):233-43.
10. Zhong G, Wang Y, Tao T, Ying J, Zhao Y. Daytime napping and mortality from all causes, cardiovascular disease, and cancer: a meta-analysis of prospective cohort studies. Sleep medicine. 2015;16(7):811-9.
11. Hayley AC, Williams LJ, Kennedy GA, Berk M, Brennan SL, Pasco JA. Excessive daytime sleepiness and metabolic syndrome: a cross-sectional study. Metabolism. 2015;64(2):244-52.
12. Fang W, Li Z, Wu L, Cao Z, Liang Y, Yang H, et al. Longer habitual afternoon napping is associated with a higher risk for impaired fasting plasma glucose and diabetes mellitus in older adults: results from the Dongfeng–Tongji cohort of retired workers. Sleep medicine. 2013;14(10):950-4.
13. Tsai TC, Wu JS, Yang YC, Huang YH, Lu FH, Chang CJ. Long sleep duration associated with a higher risk of increased arterial stiffness in males. Sleep. 2014;37(8):1315-20.
14. Abe T, Aoki T, Yata S, Okada M. Sleep duration is significantly associated with carotid artery atherosclerosis incidence in a Japanese population. Atherosclerosis. 2011;217(2):509-13.
15. Yoshioka E, Saijo Y, Kita T, Okada E, Satoh H, Kawaharada M, et al. Relation between self-reported sleep duration and arterial stiffness: a cross-sectional study of middle-aged Japanese civil servants. Sleep. 2011;34(12):1681-6.
16. Wolff B, Volzke H, Schwahn C, Robinson D, Kessler C, John U. Relation of self-reported sleep duration with carotid intima-media thickness in a general population sample. Atherosclerosis. 2008;196(2):727-32.
17. Nakazaki C, Noda A, Koike Y, Yamada S, Murohara T, Ozaki N. Association of insomnia and short sleep duration with atherosclerosis risk in the elderly. American journal of hypertension. 2012;25(11):1149-55.
18. Lennon LT, Ramsay SE, Papacosta O, Shaper AG, Wannamethee SG, Whincup PH. Cohort Profile Update: The British Regional Heart Study 1978-2014: 35 years follow-up of cardiovascular disease and ageing. International journal of epidemiology. 2015;44(3):826-g.
19. Ramsay SE, Arianayagam DS, Whincup PH, Lennon LT, Cryer J, Papacosta AO, et al. Cardiovascular risk profile and frailty in a population-based study of older British men. Heart. 2015;101(8):616-22.

For peer review only - http://bmjopen.bmj.com/site/about/guidelines.xhtml

20. Wannamethee SG, Lowe GD, Whincup PH, Rumley A, Walker M, Lennon L. Physical activity and hemostatic and inflammatory variables in elderly men. Circulation. 2002;105(15):1785-90.
21. Wannamethee SG, Whincup PH, Shaper AG, Rumley A, Lennon L, Lowe GDO. Circulating inflammatory and hemostatic biomarkers are associated with risk of myocardial infarction and coronary death, but not angina pectoris, in older men. Journal of Thrombosis and Haemostasis. 2009;7(10):1605-11.
22. Wannamethee GS, Welsh P, Whincup PH, Lennon L, Papacosta O, Sattar N. N-terminal pro brain natriuretic peptide but not copeptin improves prediction of heart failure over other routine clinical risk parameters in older men with and without cardiovascular disease: population-based study. European Journal of Heart Failure. 2014;16(1):25-32.
23. National Institute for Clinical Excellence. Chronic heart failure: the management of adults with chronic heart failure in primary and secondary care (partial update). (Clinical guideline 108) 2010 [Available from: http://www.nice.org.uk/CG108.
24. Levey AS, Stevens LA, Schmid CH, et al. A new equation to estimate glomerular filtration rate. Annals of Internal Medicine. 2009;150(9):604-12.
25. Dijk JM, Algra A, van der Graaf Y, Grobbee DE, Bots ML. Carotid stiffness and the risk of new vascular events in patients with manifest cardiovascular disease. The SMART study. European heart journal. 2005;26(12):1213-20.
26. Leng Y, Ahmadi-Abhari S, Wainwright NWJ, Cappuccio FP, Surtees PG, Luben R, et al. Daytime napping, sleep duration and serum C reactive protein: a population-based cohort study. BMJ Open. 2014;4(11):e006071.
27. Kociol RD, Pang PS, Gheorghiade M, Fonarow GC, O'Connor CM, Felker GM. Troponin Elevation in Heart FailurePrevalence, Mechanisms, and Clinical Implications. Journal of the American College of Cardiology. 2010;56(14):1071-8.
28. Kragelund C, Grønning B, Køber L, Hildebrandt P, Steffensen R. N-Terminal Pro–B-Type Natriuretic Peptide and Long-Term Mortality in Stable Coronary Heart Disease. New England Journal of Medicine. 2005;352(7):666-75.
29. Sato Y, Fujiwara H, Takatsu Y. Biochemical markers in heart failure. Journal of cardiology. 2012;59(1):1-7.
30. Laugsand LE, Strand LB, Platou C, Vatten LJ, Janszky I. Insomnia and the risk of incident heart failure: a population study. European heart journal. 2014;35(21):1382-93.
31. Ingelsson E, Lind L, Ärnlöv J, Sundström J. Sleep disturbances independently predict heart failure in overweight middle-aged men. European Journal of Heart Failure. 2007;9(2):184-90.

For peer review only

For peer review only - http://bmjopen.bmj.com/site/about/guidelines.xhtml

STROBE Statement—checklist of items that should be included in reports of observational studies

	Item No	Recommendation
**Title and abstract**	1	(*a*) Indicate the study's design with a commonly used term in the title or the abstract – **PAGE 1**
		(*b*) Provide in the abstract an informative and balanced summary of what was done and what was found – **PAGE 2**
**Introduction**		
Background/rationale	2	Explain the scientific background and rationale for the investigation being reported – **PAGE 4**
Objectives	3	State specific objectives, including any prespecified hypotheses - **PAGE 4**
**Methods**		
Study design	4	Present key elements of study design early in the paper - **PAGE 5-7**
Setting	5	Describe the setting, locations, and relevant dates, including periods of recruitment, exposure, follow-up, and data collection - **PAGE 5-7**
Participants	6	(*a*) *Cohort study*—Give the eligibility criteria, and the sources and methods of selection of participants. Describe methods of follow-up – **PAGE 5**<br>*Case-control study*—Give the eligibility criteria, and the sources and methods of case ascertainment and control selection. Give the rationale for the choice of cases and controls<br>*Cross-sectional study*—Give the eligibility criteria, and the sources and methods of selection of participants
		(*b*) *Cohort study*—For matched studies, give matching criteria and number of exposed and unexposed – **N/A**<br>*Case-control study*—For matched studies, give matching criteria and the number of controls per case
Variables	7	Clearly define all outcomes, exposures, predictors, potential confounders, and effect modifiers. Give diagnostic criteria, if applicable – **PAGE 5-7**
Data sources/ measurement	8*	For each variable of interest, give sources of data and details of methods of assessment (measurement). Describe comparability of assessment methods if there is more than one group – **PAGE 5-7**
Bias	9	Describe any efforts to address potential sources of bias – **PAGE 13**
Study size	10	Explain how the study size was arrived at - **PAGE 5**
Quantitative variables	11	Explain how quantitative variables were handled in the analyses. If applicable, describe which groupings were chosen and why – **PAGE 5-7**
Statistical methods	12	(*a*) Describe all statistical methods, including those used to control for confounding - **PAGE 7**
		(*b*) Describe any methods used to examine subgroups and interactions – **PAGE 7**
		(*c*) Explain how missing data were addressed – **PAGE 5-6**
		(*d*) *Cohort study*—If applicable, explain how loss to follow-up was addressed – **N/A**<br>*Case-control study*—If applicable, explain how matching of cases and controls was addressed<br>*Cross-sectional study*—If applicable, describe analytical methods taking account of sampling strategy
		(*e*) Describe any sensitivity analyses – **N/A**

Continued on next page

For peer review only - http://bmjopen.bmj.com/site/about/guidelines.xhtml

**Results**		
Participants	13*	(a) Report numbers of individuals at each stage of study—eg numbers potentially eligible, examined for eligibility, confirmed eligible, included in the study, completing follow-up, and analysed – **PAGE 5-6**
		(b) Give reasons for non-participation at each stage – **PAGE 5**
		(c) Consider use of a flow diagram – **N/A**
Descriptive data	14*	(a) Give characteristics of study participants (eg demographic, clinical, social) and information on exposures and potential confounders – **PAGE 7-8, 15**
		(b) Indicate number of participants with missing data for each variable of interest – **PAGE 15-19**
		(c) *Cohort study*—Summarise follow-up time (eg, average and total amount) – **PAGE 5**
Outcome data	15*	*Cohort study*—Report numbers of outcome events or summary measures over time – **PAGE 15-19**
		Case-control study—Report numbers in each exposure category, or summary measures of exposure
		Cross-sectional study—Report numbers of outcome events or summary measures
Main results	16	(*a*) Give unadjusted estimates and, if applicable, confounder-adjusted estimates and their precision (eg, 95% confidence interval). Make clear which confounders were adjusted for and why they were included – **PAGE 15-19**
		(*b*) Report category boundaries when continuous variables were categorized – **N/A**
		(*c*) If relevant, consider translating estimates of relative risk into absolute risk for a meaningful time period – **N/A**
Other analyses	17	Report other analyses done—eg analyses of subgroups and interactions, and sensitivity analyses – **PAGE 9-10**
**Discussion**		
Key results	18	Summarise key results with reference to study objectives – **PAGE 7-10**
Limitations	19	Discuss limitations of the study, taking into account sources of potential bias or imprecision. Discuss both direction and magnitude of any potential bias – **PAGE 13**
Interpretation	20	Give a cautious overall interpretation of results considering objectives, limitations, multiplicity of analyses, results from similar studies, and other relevant evidence – **PAGE 11-13**
Generalisability	21	Discuss the generalisability (external validity) of the study results – **PAGE 13**
**Other information**		
Funding	22	Give the source of funding and the role of the funders for the present study and, if applicable, for the original study on which the present article is based – **PAGE 14**

*Give information separately for cases and controls in case-control studies and, if applicable, for exposed and unexposed groups in cohort and cross-sectional studies.

Note: An Explanation and Elaboration article discusses each checklist item and gives methodological background and published examples of transparent reporting. The STROBE checklist is best used in conjunction with this article (freely available on the Web sites of PLoS Medicine at http://www.plosmedicine.org/, Annals of Internal Medicine at http://www.annals.org/, and Epidemiology at http://www.epidem.com/). Information on the STROBE Initiative is available at www.strobe-statement.org.

For peer review only

For peer review only - http://bmjopen.bmj.com/site/about/guidelines.xhtml
